# Lattice defects induced by microtubule-stabilizing agents exert a long-range effect on microtubule growth by promoting catastrophes

**DOI:** 10.1073/pnas.2112261118

**Published:** 2021-12-16

**Authors:** Ankit Rai, Tianyang Liu, Eugene A. Katrukha, Juan Estévez-Gallego, Szymon W. Manka, Ian Paterson, J. Fernando Díaz, Lukas C. Kapitein, Carolyn A. Moores, Anna Akhmanova

**Affiliations:** ^a^Cell Biology, Neurobiology and Biophysics, Department of Biology, Faculty of Science, Utrecht University 3584 CH Utrecht, the Netherlands;; ^b^Institute of Structural and Molecular Biology, Birkbeck, University of London, WC1E 7HX London, United Kingdom;; ^c^Structural and Chemical Biology, Centro de Investigaciones Biológicas Margarita Salas, Consejo Superior de Investigaciones Científicas 28040 Madrid, Spain;; ^d^Yusuf Hamied Department of Chemistry, University of Cambridge, Cambridge CB2 1EW, United Kingdom

**Keywords:** microtubule, Taxol, protofilament, in vitro reconstitution, photoablation

## Abstract

Microtubules are dynamic cytoskeletal polymers that spontaneously switch between phases of growth and shrinkage. The probability of transitioning from growth to shrinkage, termed catastrophe, increases with microtubule age, but the underlying mechanisms are poorly understood. Here, we set out to test whether microtubule lattice defects formed during polymerization can affect growth at the plus end. To generate microtubules with lattice defects, we used microtubule-stabilizing agents that promote formation of polymers with different protofilament numbers. By employing different agents during nucleation of stable microtubule seeds and the subsequent polymerization phase, we could reproducibly induce switches in protofilament number and induce stable lattice defects. Such drug-induced defects led to frequent catastrophes, which were not observed when microtubules were grown in the same conditions but without a protofilament number mismatch. Microtubule severing at the site of the defect was sufficient to suppress catastrophes. We conclude that structural defects within the microtubule lattice can exert effects that can propagate over long distances and affect the dynamic state of the microtubule end.

## Significance

Microtubules are major cytoskeletal filaments important for cell division, growth, and differentiation. Microtubules can rapidly switch between phases of growth and shortening, and this dynamic behavior is essential for shaping microtubule arrays. To obtain insights into mechanisms controlling microtubule dynamics, here we used microtubule-stabilizing agents such as Taxol and their fluorescent analogs to manipulate microtubule protofilament number and generate stable defects in microtubule lattices that can be visualized using fluorescence microscopy. We show that microtubule polymerization rate increases with protofilament number and that drug-induced microtubule lattice discontinuities can promote plus-end catastrophes at a distance of several micrometers. Our data indicate that structural defects in the microtubule wall can have long-range propagating effects on microtubule tip dynamics.

Microtubules are cytoskeletal polymers that rapidly switch between phases of growth and shortening, and this behavior, termed dynamic instability, plays a crucial role in the formation, maintenance, and reorganization of microtubule arrays during cell division, migration, and differentiation (1, 2). The transition from growth to shrinkage, an event called catastrophe, is known to occur when the protective cap of guanosine triphosphate (GTP)–bound tubulin subunits is reduced or lost, but the underlying mechanisms are still the subject of investigation (3, 4). One interesting property of microtubules is that the frequency of catastrophes depends on microtubule age: Microtubules that are growing for a longer time have a higher chance to switch to depolymerization (5, 6). Changes occurring at the microtubule end, such as loss of individual protofilaments or end tapering, have been shown to promote catastrophe (7–9). In principle, it is also possible that the catastrophe frequency at the plus end is affected by structural features in the microtubule lattice farther away from the tip, but this possibility has so far remained untested.

Structural studies have established that tubulin can form tubes with different protofilament numbers (10), dependent on the species, nucleation template, presence of different microtubule-associated proteins, and other properties of the polymerization reaction (e.g., ref. 11; reviewed in ref. 12). An important consequence of the structural plasticity of the microtubule lattice (13) is the formation of lattice defects, such as sites where a microtubule gains or loses one or more protofilaments (11, 14–17). A recent cryoelectron tomography analysis showed that in some cell types, such as *Drosophila* neurons, variations and transitions in protofilament number are readily detectable (18) and are thus likely to be physiologically relevant. Switches in protofilament number can be introduced during microtubule growth, and their presence may affect microtubule dynamics in different ways. For example, defects can be repaired through tubulin incorporation, and the resulting islands of GTP-tubulin can trigger microtubule rescue (19–22). On the other hand, the presence of defects could potentially also induce catastrophes (as proposed in ref. 14), since conformational properties of the microtubule lattice might propagate over some distances (23).

To study the relationship between lattice defects and microtubule catastrophes, one should be able to directly correlate the presence of defects with the dynamics of microtubule ends. We recently found that fluorescent analogs of microtubule-stabilizing agents (MSAs) can be used to induce microtubule lattice defects that can be visualized by fluorescence microscopy. When present at low concentrations, MSAs preferentially bind to microtubule plus ends that enter a “precatastrophe” state (24), which is manifested by the gradual loss of the GTP cap and reduced recruitment of end-binding (EB) proteins that detect GTP-bound microtubule lattice (25–27). Strong accumulation of MSAs at precatastrophe microtubule ends leads to the formation of stabilized patches of microtubule lattice, where the tube is incomplete and keeps incorporating GTP-tubulin but is not fully repaired (24). When microtubules switch to depolymerization, such persistent lattice defects, which coincide with the hotspots of MSA binding, can induce repeated rescues and, therefore, they were termed “stable rescue sites” (24).

Here, we used MSA-induced lattice defects to address two questions. First, what prevents complete repair of an MSA-induced persistent lattice defect? And second, does the presence of such a persistent defect affect the dynamics of the microtubule plus end? Since different MSAs are known to affect the number of protofilaments (15, 28–33), we hypothesized that persistent lattice defects could be associated with the changes in protofilament number and thus could not be fully repaired for geometrical reasons. We tested this idea by generating stable microtubule seeds with one MSA and then elongating them in the presence of another MSA, with the same or different preference for protofilament number. Use of fluorescent MSAs allowed us to directly follow drug binding. We found that precatastrophe microtubule ends accumulated MSAs in all conditions; however, the outcome of drug binding was different. When there was no mismatch in protofilament number between the seeds and the elongation conditions, drug accumulations were short in duration and length, and microtubule growth beyond such sites was processive. In contrast, when, based on the MSA properties, a mismatch in protofilament number could be expected, large and persistent drug accumulations were formed. The existence of such mismatches was confirmed by cryoelectron microscopy (cryo-EM) and by measuring microtubule growth rate, which became higher with increasing protofilament number. When microtubule ends extended beyond a mismatch-containing lattice defect, they displayed elevated catastrophe frequency. Laser-mediated severing of a microtubule at the site of the persistent defect reduced catastrophe frequency at the plus end. Our data demonstrate that local perturbations in microtubule structure can affect the state of the dynamic end at a distance of several micrometers.

## Results

### Protofilament Number Affects Microtubule Growth Rate.

**Fig. 1.**
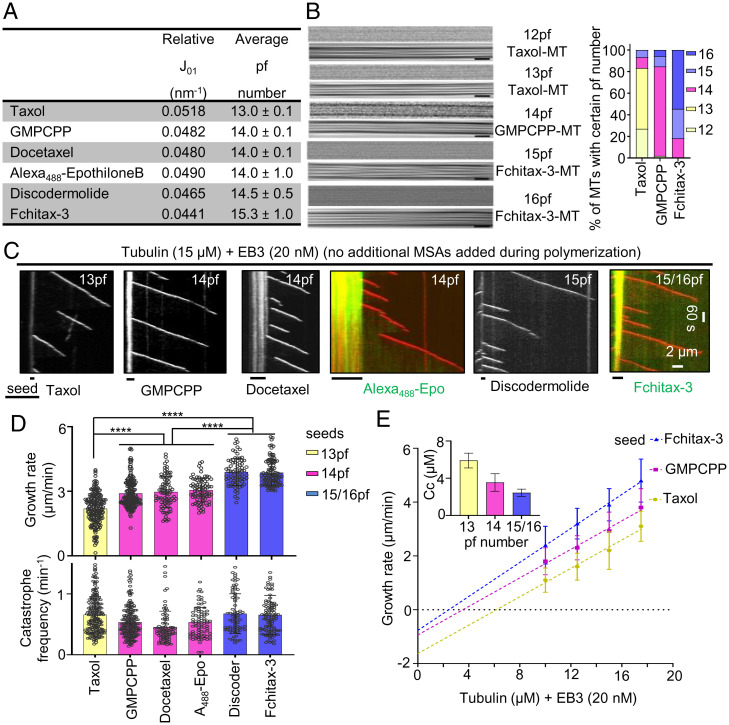
Microtubule protofilament number affects microtubule growth rate. (*A*) X-ray fiber diffraction measurements for protofilament numbers of microtubules polymerized in the presence of different MSAs. For each condition, a total of 24 diffraction images were averaged and background-subtracted using ImageJ software. (*B*) Representative raw cryo-EM images and their filtered versions emphasizing their Moiré patterns. (Scale bars, 25 nm.) The bar graph shows microtubule (MT) protofilament number distribution determined by Moiré pattern visualization for each microtubule. Microtubule population: *n* = 89 for Taxol; *n* = 52 for GMPCPP; and *n* = 77 for Fchitax-3. (*C*) Representative kymographs showing microtubule dynamics in the presence of seeds stabilized with the indicated compounds, supplemented with tubulin (15 μM) and mCherry-EB3 (20 nM) in the absence of any additional MSAs during the reaction. (*D*) Quantification of growth rates (*Upper*) and catastrophe frequencies (*Lower*) in the presence of seeds with different protofilament numbers. From left to right, *n* = 193, 196, 82, 81, 76, and 104 growth events; *n* = 2 independent experiments for docetaxel, Alexa_488_-epothilone B, and discodermolide; *n* = 3 independent experiments for Taxol, GMPCPP, and Fchitax-3. Error bars represent SD; ****P < 0.0001, Mann–Whitney *U* test. (*E*) Microtubule growth rate as a function of tubulin concentration (10 to 17.5 μM) from seeds with different protofilament numbers. Error bars represent SD; critical concentration *C*_c_ (mean ± SEM), calculated based on the linear fits of the data, is shown (*Inset*); *n* = 3 independent experiments. Microtubule growth events: *n* = 280, 161, and 221 for 10 μM; *n* = 369, 254, and 193 for 12.5 μM; *n* = 193, 196, and 104 for 15 μM; and *n* = 243, 208, and 214 for 17.5 μM for Taxol, GMPCPP, and Fchitax-3, respectively.

We first set out to test whether microtubules with different protofilament numbers display different dynamic properties. Protofilament number changes in response to the nucleotide bound to the regulatory E site or the presence of MSAs (15, 28–33). Therefore, to generate microtubules with different protofilament numbers, we prepared microtubule seeds with the slowly hydrolyzable GTP analog GMPCPP or with different microtubule-stabilizing drugs. Using X-ray fiber diffraction and cryo-EM, we confirmed previous observations showing that microtubule seeds generated in the presence of Taxol have predominantly 13 protofilaments (pf), whereas 14pf microtubules were observed in the presence of GMPCPP and docetaxel (Fig. 1 *A* and *B*) (15, 31–33). Microtubules stabilized with Alexa_488_-epothilone B also had 14pf, whereas protofilament number shifted toward 15pf in the presence of discodermolide and 15/16pf with Fchitax-3 (Fig. 1 *A* and *B*). We next used these stabilized microtubule seeds to grow dynamic microtubules and observed their behavior using total internal reflection fluorescence (TIRF) microscopy as described previously (34, 35) (*SI Appendix*, Fig. S1*A*). In these assays, the seeds contained rhodamine- and biotin-labeled tubulin for visualization and attachment to glass surface, respectively, and cryo-EM showed that in these conditions, Taxol-stabilized seeds predominantly contained 13pf, GMPCPP-stabilized seeds contained 14pf, and Fchitax-3–stabilized seeds contained 15/16pf (*SI Appendix*, Fig. S1*B*). Assays were performed either with tubulin alone or with the addition of mCherry-EB3, which serves as a microtubule plus-end marker and increases both the growth rate and catastrophe frequency in assays with purified tubulin (36) (Fig. 1*C* and *SI Appendix*, Fig. S1*C*). In these assays, MSAs were used to prepare stable seeds but were not added during polymerization. Both with and without EB3, we found that microtubule growth rate increased with protofilament number (Fig. 1 *D*, *Upper* and *SI Appendix*, Fig. S1 *D*, *Upper*). Calculation of the critical concentration *C*_c_ of microtubule polymerization and tubulin association rate constant *k*_on_ based on fitting of the dependence of the growth rate on tubulin concentration (37) indicated that *C*_c_ is lower and *k*_on_ is higher for microtubules with more protofilaments (Fig. 1*E* and *SI Appendix*, Fig. S1*E*). Catastrophe frequency showed some variability between different conditions but was independent of protofilament number (Fig. 1 *D*, *Lower* and *SI Appendix*, Fig. S1 *D*, *Lower*). As described previously (35), very few rescues were seen when microtubules were grown from GMPCPP seeds, while occasional rescues were found when drug-stabilized seeds were used, likely because some drug molecules could diffuse from the seeds and bind to the dynamic lattice.

Importantly, in these conditions, the nature of the drug present in the seeds had no effect on the polymerization rate—for example, all microtubules grown from 14pf seeds, including GMPCPP-stabilized ones, polymerized with the same speed. To further exclude that the observed differences in growth rates were caused by the drugs diffusing from the seeds, we labeled the seeds with different protofilament numbers in different colors and grew microtubules from two types of seeds on the same coverslip (*SI Appendix*, Fig. S2*A*). In these experiments, two types of seeds were exposed to exactly the same reaction mix, including the drugs that might be present in solution. Importantly, we observed that microtubules still displayed growth rates characteristic for their protofilament number (*SI Appendix*, Fig. S2*A*). For example, in the same reaction mix, microtubules grew from Taxol-stabilized seeds (13pf) slower than from GMPCPP (14pf) seeds (*SI Appendix*, Fig. S2*A*). We also investigated whether microtubule depolymerization rate depended on protofilament number but found no clear correlation: We observed that microtubules grown from 14pf or 15/16pf seeds depolymerized at the same rate, but slower than microtubules grown from Taxol-stabilized (13pf) seeds (*SI Appendix*, Fig. S2*B*). We conclude that when the growth conditions are the same, microtubule polymerization rate can be used to infer protofilament number.

### Effects of MSAs on Microtubule Dynamics Depend on the Protofilament Number in the Seeds.

**Fig. 2.**
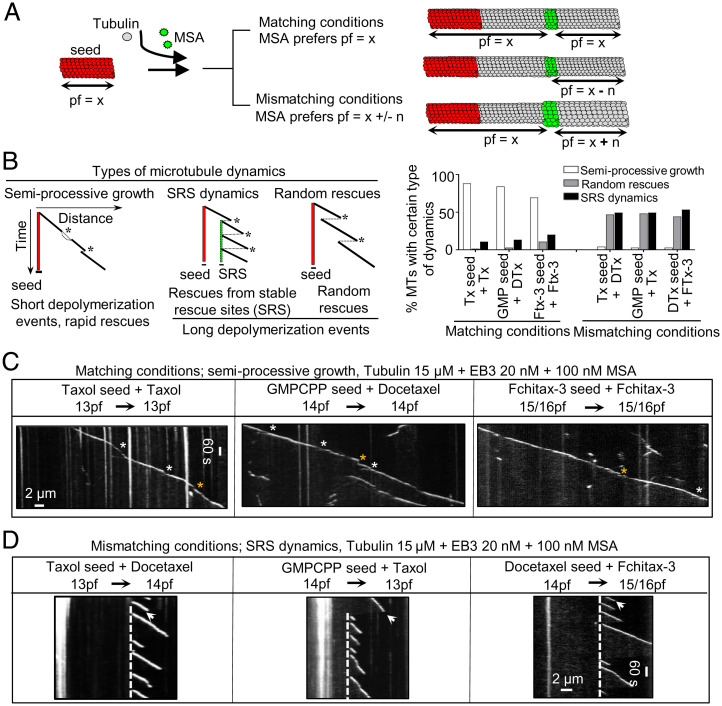
Protofilament number mismatch between the seed and growth conditions affects microtubule dynamics. (*A*) A scheme illustrating microtubule growth from seeds in the presence of MSAs with the same or different protofilament number preference. MSA-binding zones are shown in green. (*B*, *Left*) A cartoon depicting kymographs corresponding to three types of microtubule dynamics observed in different conditions. Microtubule dynamics is defined as SRS dynamics if a microtubule regrows at least three times from the same site after undergoing catastrophes. A random rescue is a single rescue event after a depolymerization episode that is longer than 0.5 μm. Asterisks highlight catastrophe events in each condition. (*B*, *Right*) Quantification of microtubule dynamics observed in the indicated conditions; *n* = 75 microtubule seeds in all conditions; *n* = 3 independent experiments. (*C* and *D*) Representative kymographs showing microtubule dynamics in the indicated conditions. In matching conditions, short growth perturbation events followed by rapid rescues are highlighted (white asterisks highlight split comets and yellow asterisks highlight depolymerization events with length 0.2 to 0.5 μm). Stable rescue sites in mismatching conditions are highlighted by white stippled lines. A white arrow highlights a long depolymerization event (>0.5 μm); *n* = 3 independent experiments.

Next, we investigated how microtubule dynamics would be affected by adding during polymerization an MSA with a protofilament number preference that was either the same (matching conditions) or different (mismatching conditions) from the one used during seed preparation (Fig. 2*A*). Drug concentrations in the range 50 to 400 nM were used, because higher concentrations induced spontaneous microtubule nucleation, and we thus could not ensure that all observed microtubules grew from the preexisting seeds with the known protofilament number. We observed three types of dynamic microtubule behaviors. The first type of dynamics was semiprocessive growth interrupted by short (0.2- to 0.5-µm) depolymerization events followed by rapid rescues (“semiprocessive growth”; Fig. 2 *B* and *C* and *SI Appendix*, Fig. S3 *A* and *C*). The second type of dynamic behavior was manifested by frequent catastrophes followed by long (>0.5-µm) depolymerization events and repeated rescues at the same site (stable rescue site, which will be termed here “SRS dynamics”; Fig. 2 *B* and *D* and *SI Appendix*, Fig. S3 *B* and *C*). The third type of dynamic behavior was characterized by catastrophes followed by long (>0.5-µm) depolymerization events and randomly distributed rescues (termed “random rescues”; Fig. 2*B* and *SI Appendix*, Fig. S4*A*). At 100 nM MSA concentrations, there was a clear difference in the occurrence of a particular type of dynamics, which depended on the combination of MSAs used for preparing the seeds and their elongation. When the protofilament number was expected to be the same (“matching conditions”; Fig. 2*A*), semiprocessive growth with very short depolymerization events strongly predominated (Fig. 2 *B* and *C* and *SI Appendix*, Fig. S3 *A* and *C*; short growth perturbations are highlighted by asterisks). In contrast, when the seeds were elongated in the presence of an MSA that had a protofilament number preference that was different from that of the MSA used for seed stabilization (“mismatching conditions”; Fig. 2*A*), SRS dynamics (highlighted by white dashed lines) or random rescues (highlighted by yellow arrows) with long depolymerization events were observed (Fig. 2 *B* and *D* and *SI Appendix*, Figs. S3 *B* and *C* and S4*A*). When higher MSA concentrations were used during microtubule growth, random rescues were predominantly observed for mismatching conditions (*SI Appendix*, Fig. S4*A*), likely because rescues became more frequent, and depolymerization events were thus not long enough to reach the preceding stable rescue site. When MSA concentration was reduced, some microtubules displayed random rescues in matching conditions, because depolymerization events became longer but were typically still followed by rapid rescues (*SI Appendix*, Fig. S4*B*).

Importantly, microtubules grown in the presence of the same MSA showed very different dynamics depending on the seeds used. For example, microtubules grown in the presence of 100 nM Taxol displayed semiprocessive growth when extending from Taxol-stabilized seeds (13pf) but SRS dynamics or random rescues when grown from GMPCPP-stabilized (14pf) or Fchitax-3–stabilized (15/16pf) seeds (Fig. 2 *B–D* and *SI Appendix*, Fig. S3). In contrast, in the presence of docetaxel, microtubules grew semiprocessively from GMPCPP or docetaxel-stabilized (14pf) seeds but showed SRS dynamics or random rescues when grown from Taxol- or Fchitax-3–stabilized seeds (Fig. 2 *B–D* and *SI Appendix*, Fig. S3). Similar results were also obtained when microtubules were grown without mCherry-EB3, although the absence of a plus-end marker made the detection of short depolymerization events less reliable (*SI Appendix*, Fig. S5). For subsequent analyses, we therefore focused on the data obtained with MSA concentrations of 100 nM in the presence of 20 nM mCherry-EB3. Together, these results demonstrate that the (mis)match between the protofilament number of the seed and the number preferred by the MSA present during elongation has a strong effect on microtubule growth dynamics, even when the microtubule tip is far away from the seed.

### Larger and More Persistent Drug Accumulations Are Observed in Mismatching Conditions.

**Fig. 3.**
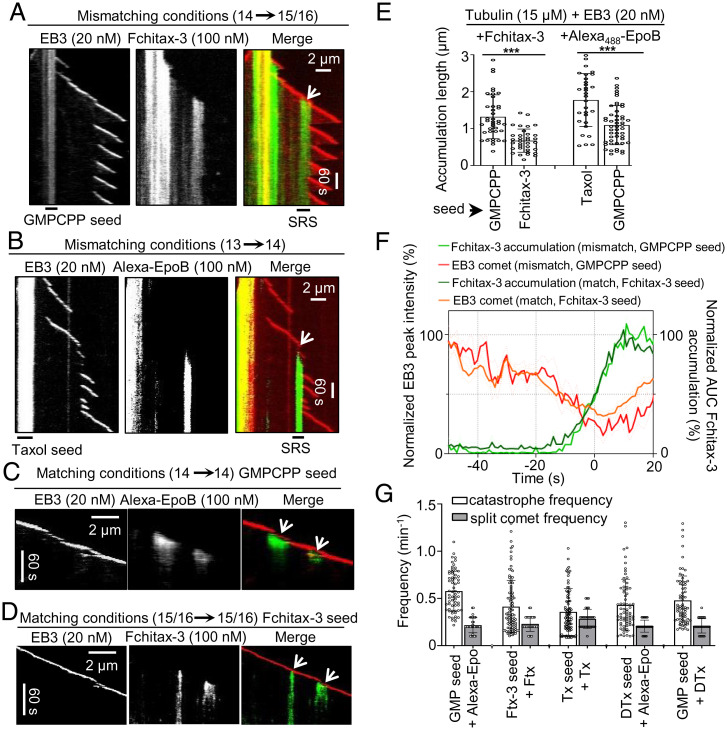
Extent of MSA accumulation at precatastrophe microtubule tips depends on the match between the seed and growth conditions. (*A* and *B*) Representative kymographs illustrating drug accumulations in mismatching conditions. Microtubules were grown from GMPCPP seeds (*A*) or Taxol seeds (*B*) in the presence of 15 μM tubulin and 20 nM mCherry-EB3 with Fchitax-3 (100 nM) (*A*) or Alexa_488_-epothilone B (100 nM) (*B*). White arrows indicate stable rescue sites with drug accumulations. (*C* and *D*) Representative kymographs illustrating drug accumulations in matching conditions. Microtubules were grown from GMPCPP seeds (*C*) or Fchitax-3 seeds (*D*) in the presence of 15 μM tubulin and 20 nM mCherry-EB3 with Alexa_488_-epothilone B (100 nM) (*C*) or Fchitax-3 (100 nM) (*D*). Split comets are indicated with white arrows. (*E*) Quantification of drug accumulation lengths in the indicated conditions. Error bars represent SD; from left to right, *n* = 39, 34, 30, and 50; *n* = 3 independent experiments; ***P < 0.001. (*F*) Time plot of averaged normalized maximum intensity of fitted EB3 comet (orange and red) and normalized area under the curve (AUC) of fitted Fchitax-3 (light and dark green) intensity profiles in mismatching conditions (as shown in *A*; *n* = 9 kymographs from five experiments) and in matching conditions (as shown in *D*; *n* = 38 kymographs from five experiments). Individual curves were aligned by maximizing cross-correlation between Fchitax-3 time curves. Error bars represent SEM. (*G*) Frequencies of catastrophes (calculated as the frequency of all growth perturbations including split comet events) and split comet events in different matching conditions. From left to right, *n* = 65, 100, 91, 71, and 70 for catastrophe frequencies for the indicated conditions and *n* = 60, 66, 73, 62, and 63 catching-up events from 30 microtubules for split comet frequencies; *n* = 3 independent experiments. Error bars represent SD.

To better understand the origin of the striking differences in the observed microtubule dynamics in matching and mismatching conditions, we visualized drug binding using fluorescent drug analogs. Stable rescue sites coincide with the formation of drug accumulation hotspots, which initiate directly behind a growing microtubule plus end entering a precatastrophe state that can be distinguished by the reduction in EB3 signal (24) (Fig. 3 *A* and *B*). Microtubule lattice zones with high drug affinity can extend for several micrometers but then abruptly stop (Fig. 3 *A* and *B*), and previous work suggested that they might represent incomplete tubes, which stop binding the drug when they close (24).

In mismatching conditions, such as GMPCPP seeds (14pf) elongated in the presence of Fchitax-3 (15/16pf) or Taxol-stabilized seeds (13pf) elongated in the presence of Alexa_488_-epothilone B (14pf), we observed large and persistent drug accumulation zones that coincided with stable rescue sites (Fig. 3 *A* and *B*). In contrast, in matching conditions (GMPCPP-stabilized seeds elongated in the presence of Alexa_488_-epothilone B [14pf] or Fchitax-3–stabilized seeds elongated in the presence of Fchitax-3 [15/16pf]), drug accumulation patches were short in duration and length (Fig. 3 *C–E* and *SI Appendix*, Fig. S6*A*). In both conditions, drug binding always initiated directly behind a microtubule plus end after it started to lose mCherry-EB3 signal, indicating a growth perturbation. The kinetics of the reduction of EB3 signal before drug binding showed considerable variability but was similar in matching and mismatching conditions (Fig. 3*F*), indicating that in both situations, microtubules could enter a precatastrophe state. The initial phase of drug accumulation was also very similar for matching and mismatching conditions (Fig. 3*F*).

Importantly, growth perturbations in matching conditions were typically of limited duration and were often accompanied by the emergence of a second, faster comet at the rear of the drug accumulation site (Fig. 3 *C* and *D* and *SI Appendix*, Fig. S6*A*). Previous work showed that a “catching-up” comet appears when some protofilaments at the growing microtubule tip are stalled whereas the others keep elongating. When the stalled protofilaments start to regrow, a faster rear comet emerges and ultimately fuses with the leading one (8, 38). We observed split comets during growth perturbations in all tested matching conditions, and also when nonfluorescent MSAs were used (*SI Appendix*, Fig. S6 *B* and *C*). Clear split comets were seen in 38 to 79% of all catastrophe events detected in matching conditions (Fig. 3*G*; all growth perturbations with length >0.2 µm, including split comet events, were considered as catastrophes, as indicated by asterisks in Fig. 2*B*). Since the two comets must be located at a significant distance from each other to be registered as a “split comet” by fluorescence microscopy, these numbers are likely underestimates of the actual frequency of such events. We conclude that in matching conditions, growth perturbations are followed by the formation of catching-up comets, which likely help to restore a normally growing microtubule plus end, leading to semiprocessive microtubule growth.

In mismatching conditions, drug accumulation zones were longer and much more persistent (Fig. 3 *A–E*). The binding density of Fchitax-3 in mismatching conditions [approximately one or two drug molecules per 8 nm (24)] was higher than in matching conditions (0.3 to 0.9 molecules per 8-nm microtubule length; *SI Appendix*, Fig. S6*D*). This can be explained by the fact that in mismatching conditions, drug accumulations expand for a longer time, possibly because a normal tube is more difficult to restore and protofilaments continue growing as a sheet or some other microtubule end structure that promotes drug binding. Thus, a mismatch between the protofilament number preference of the MSA used to prepare the seeds and to elongate them inhibits the restoration of a growing microtubule plus end after a growth perturbation has occurred and a drug accumulation has formed.

### Lattice Defects Observed in Mismatching Conditions Are Associated with Switching of Protofilament Number.

**Fig. 4.**
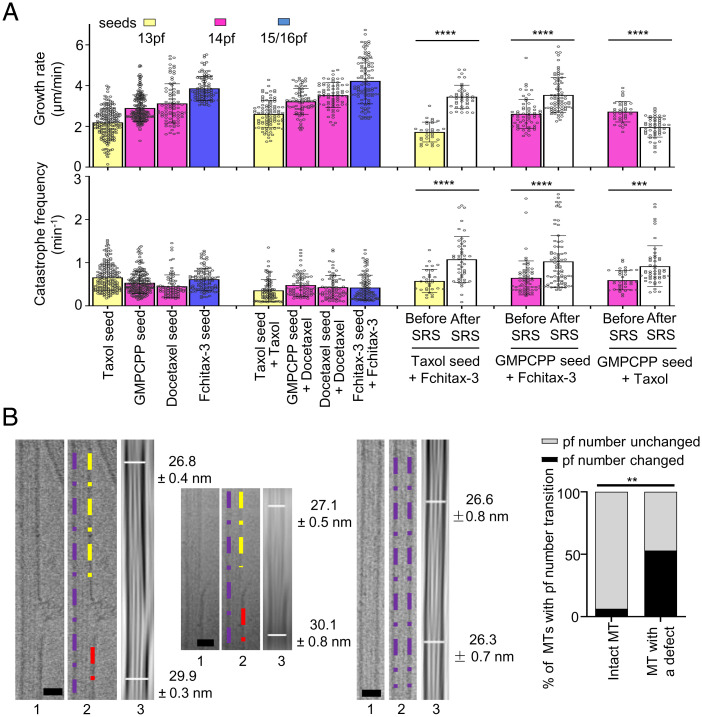
Lattice defects observed in mismatching conditions are associated with switching of protofilament number. (*A*) Quantification of growth rates (*Upper*) and catastrophe frequencies (*Lower*; calculated as the frequency of all growth perturbations including catching-up events) for the indicated conditions. For better comparison, data from Fig. 1*D* are presented again in the present panels; *n* = 193, 196, 82, and 104 microtubule growth events for microtubules polymerized from Taxol, GMPCPP, docetaxel, and Fchitax-3 seeds; *n* = 91, 70, 71, and 100 in the presence of Taxol seed + Taxol (100 nM), GMPCPP seed + docetaxel (100 nM), docetaxel seed + docetaxel (100 nM), and Fchitax-3 seed + Fchitax-3 (100 nM); *n* = 30 and 40 for Taxol seed + Fchitax-3 (100 nM) before and after SRS formation; *n* = 60 and 75 for GMPCPP seed + Fchitax-3 (100 nM) before and after SRS formation; *n* = 35 and 51 for GMPCPP seed + Taxol (100 nM) before and after SRS formation; *n* = 3 independent experiments. Error bars represent SD. The colors of the bars and protofilament values indicate the protofilament number preferences of a particular drug used. ***P < 0.001, ****P < 0.0001, Mann–Whitney *U* test. (*B*) Representative raw cryo-EM images and their filtered versions with enhanced microtubule Moiré patterns showing the diameter difference (protofilament number transition) for microtubules grown from GMPCPP seeds in the presence of Fchitax-3 (mismatching condition; Fig. 3*A*). For each panel, *Left* and *Central* show defect-containing microtubules and *Right* shows a microtubule with no visible defects. (Scale bars, 25 nm.) Images 1 and 2 are raw cryo-EM images, with purple, yellow, and red dashed lines highlighting diameter differences on either side of a defect in image 2. Image 3 shows the diameters for two ends of a microtubule. Diameters were measured in Fiji. Percentage of microtubules with protofilament number transition in microtubules with no visible defects (*n* = 19) and microtubules with sheet-like defects (*n* = 17). The percentage differences were evaluated by two-sided Fisher’s exact test; **P = 0.0023.

We hypothesized that binding of the drug to a precatastrophe microtubule tip either induces or stabilizes tubes with a protofilament number fitting with the specific preference of the drug used. In mismatching conditions, this would cause a protofilament number switch occurring at the stable rescue site, and this could explain why such sites do not get fully repaired. If this hypothesis is correct, a microtubule will be expected to grow with the speed characteristic for the number of protofilaments present in the seed before the stable rescue site, but with a speed characteristic for the MSA present in the growth reaction after it. We found that in matching conditions, the addition of any MSA caused an increase in microtubule growth rate, but the correlation between the rate and protofilament number was retained (Fig. 4 *A*, *Upper*). Interestingly, microtubules with SRS dynamics in mismatching conditions displayed growth rates characteristic for the seed before the rescue site and the growth rate matching better that of the MSA used in the growth reaction after the stable rescue site. For example, when GMPCPP seeds (14pf) were elongated in the presence of Fchitax-3 (15/16pf) or Taxol (13pf), the growth rate was characteristic for 14pf microtubules before the stable rescue site but was elevated after a stable rescue site in the presence of Fchitax-3 and decreased in the presence of Taxol (Fig. 4 *A*, *Upper*). In contrast, in matching conditions, growth rate before and after a growth perturbation did not change (*SI Appendix*, Fig. S7*A*). It should be noted, however, that the changes in growth rate did not completely match the speeds of microtubule growth when the same MSA was used for seed stabilization and elongation (Fig. 4 *A*, *Upper*). This likely reflects variability in protofilament number after the switch at a stable rescue site.

Further support for the occurrence of protofilament number switching at the stable rescue sites was obtained by cryo-EM. In our previous study (24), we found that microtubule lattice discontinuities corresponding to Fchitax-3 accumulations can be detected by cryo-EM. Here, we analyzed these data focusing on the microtubule diameter at the two sides of a lattice defect and found that protofilament number changed in ∼53% of such cases, whereas in microtubules lacking defects switches in protofilament number were rare (Fig. 4*B* and *SI Appendix*, Fig. S7*B*). Since not all defects detected by cryo-EM might represent stable rescue sites observed by fluorescence microscopy, this number likely represents an underestimate of the actual switching of protofilament number at stable rescue sites. Thus, MSAs present during microtubule elongation can induce a switch to their preferred protofilament number at the stable rescue site.

### Protofilament Number Mismatch between the Seed and the Growing Microtubule Lattice Promotes Catastrophes.

Having established that stable rescue sites can correspond to regions where a switch in protofilament number takes place, we next asked how such sites affect growth of microtubules extending beyond them. Interestingly, we found that whereas catastrophe frequency (calculated as the frequency of all growth perturbations >0.2 µm including catching-up events) remained constant for microtubules growing in matching conditions, it was strongly increased for the growth events occurring after the stable rescue site (Fig. 4 *A*, *Lower*). The increase in catastrophe frequency was similar for microtubules that switched to higher (e.g., from 13pf or 14pf to 15/16pf) or lower (from 14pf to 13pf) numbers of protofilaments (Fig. 4 *A*, *Lower*). This observation was surprising, because one would expect that after switching to the protofilament number “preferred” by the MSA present in solution, a microtubule will be further growing in matching conditions and should thus display semiprocessive growth without long depolymerization events. However, this was not the case: Microtubule plus ends entering a precatastrophe state after a stable rescue site typically did not accumulate MSAs and simply switched to depolymerization, which proceeded all the way back to the preceding stable rescue site (Fig. 3 *A* and *B*). Whereas 48% of all catastrophe and precatastrophe events (distinguished by strong reduction or complete loss of the EB3 signal) occurring during microtubule outgrowth from the seed led to drug accumulation and formation of a stable rescue site, only 5% of such events occurring after a stable rescue site triggered drug accumulation and microtubule stabilization (*SI Appendix*, Fig. S8*A*). Formation of a secondary stable rescue site was thus quite rare: For example, when microtubules extended from GMPCPP-stabilized seeds in the presence of Fchitax-3, the formation of secondary stable rescue sites was seen only in 11% of all observed microtubules (*SI Appendix*, Fig. S8*B*). This suggests that some properties of a precatastrophe microtubule plus end extending after a stable rescue site (after a microtubule has incorporated a lattice defect) are different from those of microtubule ends growing directly from the seed.

To explore the underlying mechanism, we analyzed the intensities of EB3 comets and microtubule tip tapering during growth episodes from the GMPCPP-stabilized seed (14pf) either in the absence of drugs (control) or after a stable rescue site induced by discodermolide (14/15pf, mismatching conditions). We note that Fchitax-3 or Alexa_488_-epothilone B could not be used in these experiments because we used green (HiLyte 488–labeled tubulin) fluorescence to obtain tubulin profiles. We focused on the 40-s time interval preceding a catastrophe in order to determine whether the precatastrophe plus ends of microtubules growing directly from the seed or from a stable rescue site are somehow different. Microtubule end tapering was similar in both conditions (*SI Appendix*, Fig. S8*C*). The reduction of EB3 signal during catastrophe onset, which is expected to reflect the kinetics of GTP cap loss during catastrophe initiation, was also quite similar, although the curve was somewhat less steep for microtubules growing from seeds compared with microtubules elongating beyond a stable rescue site (*SI Appendix*, Fig. S8*D*). A higher-resolution analysis would be needed to determine what makes precatastrophe microtubule tips growing from seed different from those elongating after a stable rescue site.

### Microtubule Severing at the Lattice Defect Site Suppresses Catastrophes.

Why do microtubules growing beyond a stable rescue site display more catastrophes? One possibility is that a lattice defect with a different number of protofilaments on the two sides affects the growth at the plus end through a long-range conformational alteration or mechanical strain. If this is the case, severing the microtubule at the site of the defect should reduce catastrophe frequency. To test this idea, we performed microtubule severing on a TIRF microscope using a pulsed 532-nm laser and observed the dynamics of the severed part of the microtubule (*SI Appendix*, Fig. S9*A*). The severed microtubule fragment was no longer attached to the coverslip. However, due to the presence of methylcellulose, which increases the viscosity of the solution and dampens fluctuations, most severed microtubule fragments did not float away but stayed close to the surface and could still be observed by TIRF microscopy (*SI Appendix*, Fig. S9*B* and Video S1). In some cases, they underwent diffusive movements; however, movements of the whole microtubule segment could be easily distinguished from microtubule growth and shortening by the synchronous displacement of fluorescent speckles present along the microtubule shaft (*SI Appendix*, Fig. S9*B*). After microtubule severing in the absence of MSAs (microtubules polymerized in the presence of GMPCPP seeds with 15 µM tubulin and 20 nM EB3), freshly generated microtubule plus ends typically depolymerized, whereas freshly generated minus ends displayed heterogeneous behavior. The poor survival of microtubules after severing and the heterogeneity in minus-end dynamics precluded a meaningful analysis of the severing data in the absence of MSAs.

**Fig. 5.**
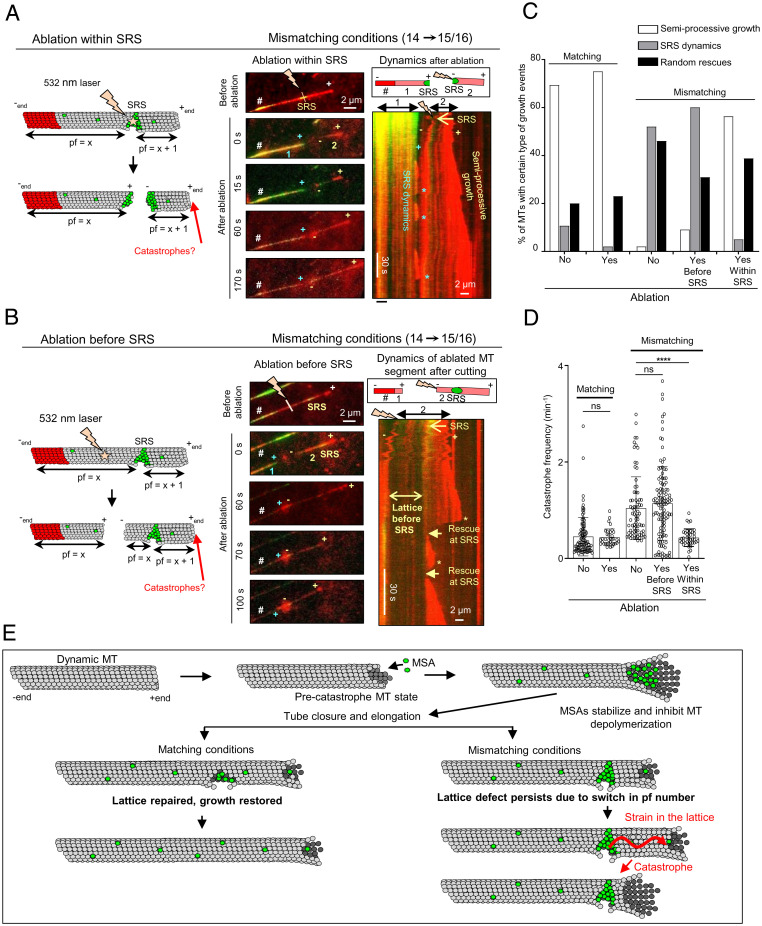
Microtubule severing at the stable rescue site suppresses catastrophes. (*A* and *B*) Schematic representation and still images showing photoablation of microtubule regions within (*A*) or before (*B*) a drug accumulation zone (SRS) and kymographs showing microtubule dynamics of the severed parts. The assay was performed in the presence of GMPCPP seeds with 15 μM tubulin supplemented with 3% rhodamine-tubulin, 20 nM mCherry-EB3, and 100 nM Fchitax-3. The time-lapse images before photoablation show the presence of stable rescue sites; the site of ablation within (*A*) and before (*B*) the stable rescue site is indicated by a lightning bolt. The newly generated microtubule fragments (1: seed-attached part; 2: the part that is detached from the seed after ablation), microtubule plus (+) ends, and the new plus (+) and minus (−) ends generated after ablation are indicated. In time-lapse images, # indicates the position of the GMPCPP seed. (*A*) The kymograph illustrates the dynamics of both fragments (1 and 2, as shown in the scheme) generated after ablation. (*B*) The kymograph illustrates the dynamics of the severed microtubule fragment (2, as shown in the scheme). Asterisks highlight catastrophes, and rescues at the stable rescue site are indicated by arrows in *B*. The labels are in blue for fragment 1 (seed-attached microtubule part) and yellow for fragment 2 (the part detached after ablation); *n* = 5 independent experiments. (*C*) Quantification of growth dynamics of the unsevered and severed microtubule segment in matching (*n* = 75, no severing; *n* = 35, after severing) and mismatching conditions; *n* = 100, 60, and 55 for no ablation, or ablation either before or within a drug accumulation area, respectively. (*D*) Quantification of catastrophe frequencies (calculated as the frequency of all growth perturbations including catching-up events). Matching conditions (Fchitax-3 seeds + 100 nM Fchitax-3): *n* = 100 (no ablation) and *n* = 35 (after ablation). Mismatching conditions (GMPCPP seeds + 100 nM Fchitax-3): *n* = 76 (no ablation, after the formation of a stable rescue site), *n* = 115 (after photoablation in a region preceding the Fchitax-3 accumulation area [SRS]), and *n* = 39 (after photoablation within SRS). ns, not significant; ****P < 0.0001, Mann–Whitney *U* test. (*E*) Model depicting microtubule lattice repair in matching and mismatching conditions. In the precatastrophe state, MSAs stabilize depolymerizing protofilaments and inhibit depolymerization. In matching conditions, this leads to rapid repair of microtubule defects and restoration of microtubule growth. In mismatching conditions, lattice defects persist despite repair due to a switch in protofilament number. The presence of a defect might generate strain in the lattice, which would affect the growing microtubule end and induce catastrophe.

In the presence of an MSA such as Fchitax-3, the severed microtubule ends were typically quite stable. To generate microtubules with defects that would be visible by fluorescence microscopy, we grew microtubules in mismatching conditions from GMPCPP-stabilized seeds (14pf) in the presence of 100 nM Fchitax-3 (15/16pf), rhodamine-labeled tubulin, and 20 nM mCherry-EB3. After photoablation, the newly generated plus end, which remained attached to the seed, as well as the new minus end and the preexisting plus end of the microtubule fragment that was detached from the seed, could all polymerize (Fig. 5*A*). Imaging before photoablation and the photoablation itself induced significant photobleaching, and the tubulin freshly incorporated at the growing microtubule ends after severing could be readily observed because its signal was brighter; growing plus ends were additionally visualized by the accumulation of mCherry-EB3, that was detected in the same fluorescent channel as tubulin (Fig. 5*A*). Even when the severed end underwent some displacement after photoablation, the plus and the minus ends could be easily distinguished from each other by their growth rate, and the remnant of the drug accumulation zone at the stable rescue site was also visible (labeled as SRS in Fig. 5*A*).

When we severed microtubules directly at the site of Fchitax-3 accumulation (a stable rescue site), a part of the drug accumulation zone at the seed-attached microtubule fragment was often preserved after ablation. The plus end outgrowing from this zone displayed frequent catastrophes, typical for the SRS dynamics (blue asterisks in Fig. 5*A* and *SI Appendix*, Fig. S10*A*). However, the catastrophe frequency at the plus end of the newly generated microtubule fragment distal from the seed (with the minus end located at the former stable rescue site) significantly decreased and a large proportion of microtubules switched from SRS dynamics with long depolymerization episodes to semiprocessive growth with short depolymerization episodes (Fig. 5 *A*, *C*, and *D*, *SI Appendix*, Fig. S10*A*, and Video S2).

In contrast, when we severed microtubules at a location preceding an Fchitax-3 accumulation zone, so that the stable rescue site with the flanking region on the minus-end side was preserved, microtubule plus ends distal from the severing site still underwent catastrophes, as was typical for the SRS dynamics (Fig. 5 *B–D*, *SI Appendix*, Fig. S10*B*, and Video S3). Further, in matching conditions, we did not observe any difference in microtubule growth pattern before and after ablation: When ablated within or before drug accumulation zones, both newly generated microtubule plus ends exhibited semiprocessive growth (Fig. 5 *C* and *D* and *SI Appendix*, Fig. S11*A*).

These data support the idea that drug-induced lattice discontinuities can exert an effect on the growth of microtubule plus ends located a significant distance away. To estimate this distance, we measured the average length of microtubule growth from the stable rescue site. This length was shorter than the average microtubule growth length after a seed, in line with the conclusion that catastrophe frequency after a stable rescue site is elevated (*SI Appendix*, Fig. S11*B*). However, microtubules could still extend from a stable rescue site for 1 to 10 µm before undergoing a catastrophe (*SI Appendix*, Fig. S11*B*). This indicates that conformational alterations or strain can propagate from a drug-induced defect to induce plus-end catastrophe at a distance encompassing hundreds of tubulin dimers.

## Discussion

In this study, we addressed how microtubule lattice defects induced by MSAs affect growth at the microtubule plus end. We made use of the fact that when microtubules are grown in the presence of nonsaturating concentrations of MSAs, such as taxanes, the drugs strongly bind to microtubule ends in a precatastrophe state and thereby induce regions of increased microtubule stability, termed stable rescue sites (24). Importantly, these sites can contain “holes” in the microtubule wall, where fresh tubulin can be incorporated (24). The exact structural nature of these defects is likely to be complex. For example, recent cryo-EM work has shown that microtubules are not perfectly cylindrical but display strong local deviations from helical symmetry with different lateral contact geometries, and these deviations can be affected by taxanes (39). Local deviations from a cylindrical shape and additional types of microtubule lattice conformations, such as tubulin sheets (40) or protofilament flares (41), will likely have a major impact on the defect structure, stability, and affinity for MSAs. Importantly, in the current study, we show that MSA-induced lattice defects represent areas of alterations in protofilament number, providing a simple geometrical reason for their persistence over time, despite continuous microtubule repair. This view is supported both by cryo-EM data and by measuring microtubule growth speeds, which we found to correlate with protofilament number.

The observation that an increase in microtubule protofilament number leads to a higher growth rate in the absence of a clear effect on the catastrophe frequency or depolymerization rate is unexpected and intriguing. In principle, one might expect that if the flux of tubulin subunits onto the end were independent of protofilament number, more protofilaments would lead to slower growth. Since we find that microtubules with more protofilaments grow faster, tubulin flux is probably not a major determinant of the microtubule growth rate in the conditions we use. Microtubules with higher protofilament number have a smaller angular separation between protofilaments, and this can affect the strength of lateral tubulin bonds and the ease with which they form. The current understanding of the molecular details of microtubule growth relies on combining experiments with modeling (42–47). For example, one recent model of microtubule polymerization assumes that growing microtubule tips have flared protofilaments, to the end of which tubulin dimers are added before the protofilaments associate with each other laterally (48). In this model, lateral interactions between tubulins were described by two parameters, an activation energy barrier and bond strength. Increasing protofilament number could reduce the lateral activation energy barrier and lead to stronger lateral bonds. According to this model, both effects would accelerate microtubule growth; however, these effects might potentially compensate each other during depolymerization, because strengthening of the lateral bonds would impede microtubule disassembly, whereas reducing the lateral activation energy barrier could promote it (48). Furthermore, the abundance of various types of lateral tubulin contacts, such as “seam-like” contacts between α- and β-tubulin, which may be weaker and potentially unfavorable for microtubule growth, can be different in microtubules with different protofilament number and can be affected by MSAs (10, 11, 39, 49, 50). Four-start microtubule lattices (15/16pf), which have no seam, may therefore grow faster. Finally, if microtubules initially grow as two-dimensional tubulin sheets (40), sheet closure might occur more readily for microtubules with more protofilaments.

Modification of protofilament number using MSAs allowed us to reproducibly generate lattice defects and explore the consequences they have for microtubule dynamics (Fig. 5*E*). Strikingly, we found that although a microtubule could polymerize beyond a persistent lattice defect for lengths up to several micrometers, microtubule plus-end dynamics were affected, as the catastrophe frequency after the defect was significantly increased (Fig. 5*E*). A striking difference between microtubule plus ends grown from defect-bearing stable rescue sites and from stabilized seeds was that the latter could be efficiently “rescued” by an MSA once they entered a precatastrophe state. Drug accumulations leading to microtubule stabilization were ∼10-fold more frequent at the precatastrophe tips growing from seeds compared with microtubule ends entering a catastrophe after a preceding stable rescue site. These data indicate that some conformational aspects of a precatastrophe plus end during growth after a seed are different from those of a plus end elongating beyond a stable rescue site, and this might reflect differences in the catastrophe induction mechanism. However, our fluorescence-based measurements detected no significant change in microtubule end tapering and only a slight difference in the decay in EB3 intensity at precatastrophe ends of microtubules growing from seeds or after a stable rescue site. It would be interesting to use cryoelectron tomography to analyze the differences between growing microtubule ends in these conditions.

Strikingly, when microtubules were growing in matching conditions (when the MSAs used to stabilize the seeds and to elongate them had the same protofilament number preference), almost every growth perturbation resulted in rapid drug binding and tip repair, leading to semiprocessive microtubule growth. These data show that the state and dynamics of the microtubule plus end depend not just on the conditions of polymerization but also on the state of the preceding lattice (Fig. 5*E*). For example, microtubules growing in the presence of 100 nM Fchitax-3 from an Fchitax-3–stabilized seed and from a GMPCPP-stabilized seed after a stable rescue site encounter exactly the same concentrations of the drug and tubulin. Yet, in the first case, microtubule polymerization is somewhat faster and semiprocessive, because although growth perturbations still occur just as in the absence of MSAs, microtubule ends transitioning to catastrophe are quickly repaired through drug binding and subsequent catching-up events. In contrast, microtubule plus ends growing in the same conditions from a stable rescue site (i.e., after a lattice defect) undergo frequent catastrophes and are not repaired through MSA binding. Therefore, in mismatching conditions, catastrophes typically evolve into long depolymerization episodes.

Importantly, microtubule severing at the site of the defect made microtubules less catastrophe-prone, in line with the view that a lattice defect at the stable rescue site has propagating properties. The underlying mechanism is unclear, but it is possible that both tubulin extension or compaction in the axial direction (51, 52) and changes in angles between protofilaments due to heterogeneity of lateral contacts (39) might play a role. One surprising feature of the drug effects in our experiments is that they can be exerted at quite low drug-binding densities (in some cases, less than one drug molecule per 8 nm of microtubule length or one tubulin “ring”). This suggests that effects of drug binding to a single tubulin dimer can propagate within the microtubule lattice. This is comparable to the reported effect of kinesin-1, where a few molecules binding to a microtubule could affect the structural properties of this microtubule (53).

The finding that lattice defects have a propagating impact on microtubule plus-end dynamics has important consequences for the concept of microtubule aging. Previous work showed that the catastrophe frequency increases when a microtubule grows for a longer time, indicating that multiple steps are needed to induce a catastrophe (5, 6, 47). However, the nature of these steps is still unclear: They may be associated with a gradual change in the microtubule end structure (e.g., more tapering) (7, 9, 42), but may also occur within the lattice. Both types of changes might play a role and, in fact, our data suggest that the mechanistic basis of catastrophe induction may differ, as precatastrophe microtubule tips can be different both in terms of drug accumulation and EB3 decay. We found that the occurrence of drug-induced lattice defects associated with protofilament number mismatch leads to catastrophe. Whether protofilament number switching occurring in the absence of MSAs can also lead to catastrophe is currently unclear and deserves further investigation. Lattice defects including switches in protofilament number have been extensively documented in previous studies (11, 14–17). Since microtubule lattice defects can accumulate over time, they could potentially contribute to microtubule aging. Repair associated with microtubule defects has been described both in vitro and in cells but, until now, microtubule lattice discontinuities have been mostly linked to the formation of GTP islands, rescues, and microtubule stabilization (19–22, 54). Here, we show that catastrophe induction is another important consequence of at least some types of lattice defects, and that lattice discontinuities can have a destabilizing effect on microtubules, a possibility that would need to be explored for defects occurring in the absence of MSAs in vitro and in cells.

It is also important to note that the drugs we employed, such as Taxol, docetaxel, and epothilone B, are either used for therapies for cancer or considered for the treatment of neurodegenerative disorders (55–57). The concentrations of the drugs used in our assays are within therapeutically relevant ranges, and the knowledge that these drugs can differentially affect microtubule dynamics based on protofilament number preferences is relevant for optimizing their therapeutic applications.

## Materials and Methods

### Analysis of Protofilament Number.

X-ray fiber diffraction images were collected on beamline BL11-NCD-SWEET of the ALBA Synchrotron. Purified bovine tubulin (5 mg) was diluted to a final concentration of 100 µM in PM buffer (80 mM Pipes, 1 mM ethylene glycol-bis[2-aminoethylether]-N,N,N′,N′-tetraacetic acid, 0.2 mM Tris, 1 mM dithiothreitol), containing 3 mM MgCl_2_, 2 mM GTP, and the corresponding compound (200 µM). Samples were incubated at 37 °C for 30 min to achieve maximum polymerization and then were mixed in a 1:1 volume ratio with prewarmed PM buffer containing 3 mM MgCl_2_ and 2% methylcellulose (MO512; Sigma-Aldrich). Final concentrations of protein, nucleotide, and compound were 50 µM tubulin, 1 mM GTP, and 100 µM compound. Samples were centrifuged for 10 s at 2,000 × *g* to eliminate air bubbles and transferred to a share-flow device (29, 58).

For each condition, 24 diffraction images were averaged and background-subtracted using ImageJ software (version 1.51j8; Wayne Rasband, NIH, Bethesda, MD). Angular image integrations were performed using XRTools software (obtained upon request from beamline BM26-DUBBLE of the European Synchrotron Radiation Facility). For average protofilament number determination, image analysis was performed as previously described (59) considering the absolute position of J_01_ for Taxol (0.0518 nm) as the reference for determining the relative peak positions for the remaining assayed conditions.

Cryo-EM was carried out as described previously (24); see *SI Appendix* for details.

### In Vitro Assays of Microtubule Dynamics and Laser Ablation.

In vitro assays for microtubule growth dynamics using TIRF microscopy and laser ablation were performed as described previously (24, 34, 35); see *SI Appendix* for experimental details and for details of image analysis. Photoablation assays were performed on a TIRF microscope which was equipped with an ILas System (Roper Scientific France/PICT-IBiSA) and a 532-nm Q-switched pulsed laser (Teem Photonics). In vitro microtubule dynamics assays were performed in the presence of GMPCPP-stabilized microtubule seeds with 15 µM tubulin supplemented with 3% rhodamine-tubulin and 20 nM mCherry-EB3 without (control) or with 100 nM Fchitax-3. Control microtubules were severed randomly by a 532-nm-focused laser beam. In mismatching conditions (GMPCPP seeds extended in the presence of 100 nM Fchitax-3), microtubule lattices were ablated at the site of Fchitax-3 accumulations or in the region between the seed and an Fchitax-3 accumulation.

## Supplementary Material

Supplementary File

Supplementary File

Supplementary File

Supplementary File

## Data Availability

The data that support the conclusions are available in the manuscript; the original fluorescence microscopy datasets are available upon request to the corresponding author. All MATLAB code, kymographs, and regions of interest used for the analysis reported in this paper have been deposited in Figshare (https://figshare.com/collections/Microtubule_lattice_defects_promote_catastrophes/5287663) (60).
