# Supplementary material for: Lattice defects induced by microtubule-stabilizing agents exert a long-range effect on microtubule growth by promoting catastrophes
Source: Proc Natl Acad Sci U S A. 2021 Dec 16;118(51):e2112261118. doi: 10.1073/pnas.2112261118 (PMC8713758; doi:10.1073/pnas.2112261118)
Supplement: Supplementary File [file pnas.2112261118.sapp.pdf]

PNAS
www.pnas.org

Supplementary Information for

Lattice defects induced by microtubule-stabilizing agents exert a long-range effect on microtubule growth by promoting catastrophes

Ankit Rai[1,5], Tianyang Liu[2], Eugene A. Katrukha[1], Juan Estévez-Gallego[3], Szymon W. Manka[2] Ian Paterson[4], J. Fernando Díaz[3], Lukas C. Kapitein[1], Carolyn A. Moores[2] and Anna Akhmanova[1#]

[1] Cell Biology, Neurobiology and Biophysics, Department of Biology, Faculty of Science, Utrecht University, Padualaan 8, 3584 CH Utrecht, the Netherlands
[2] Institute of Structural and Molecular Biology, Birkbeck, University of London, Malet Street, London, United Kingdom
[3] Structural and Chemical Biology, Centro de Investigaciones Biológicas Margarita Salas, Consejo Superior de Investigaciones Científicas CIB-MS, CSIC, Ramiro de Maeztu 9, 28040, Madrid, Spain
[4] Yusuf Hamied Department of Chemistry, University of Cambridge, Cambridge CB2 1EW, United Kingdom
[5] Current address: Department of Biochemistry, Faculty of Science, Banaras Hindu University, Varanasi, India

# Corresponding author: Anna Akhmanova,
Email: a.akhmanova@uu.nl

This PDF file includes:

Supplementary text for Materials and Methods

Figures S1 to S11 with their legends

Legends for Videos S1 to S3

SI References

Other supplementary materials for this manuscript include the following:

Video S1 to S3

Supplementary Information Text

Reagents and purified proteins

Taxol, 1,4-piperazinediethanesulfonic acid (PIPES), GTP, methylcellulose, glucose oxidase from Aspergillus niger, catalase from bovine liver, dithiothreitol (DTT), magnesium chloride, ethylene glycol-bis(2-aminoethylether)-N,N,N',N'-tetraacetic acid (EGTA), potassium chloride, potassium hydroxide, κ-casein and glucose were obtained from Sigma-Aldrich. GMPCPP was obtained from Jena Biosciences. Biotinylated poly(l-lysine)- [g]-poly(ethylene glycol) (PLL-PEG-biotin) was obtained from Susos AG. NeutrAvidin was obtained from Invitrogen. Different types of labelled and unlabeled purified tubulin used in the assays were either purchased from Cytoskeleton or purified as described previously (1) for X-ray fiber diffraction experiments.

Docetaxel was procured from Sanofi-Aventis. Fchitax-3 was provided by Wei-Shuo Fang (State Key Laboratory of Bioactive Substances and Functions of Natural Medicines, Institute of Materia Medica, Beijing, China (2). Alexa488-Epothilone B was obtained from Simon Glauser and Karl-Heinz Altman (Department of Chemistry and Applied Biosciences, Institute of Pharmaceutical Sciences, ETH Zürich, Zurich, Switzerland (3). Discodermolide was synthesized as described previously (4).

Cryo-Electron Microscopy

Microtubule polymerization: For the analysis of protofilament number distribution, microtubules in the presence of GMPCPP or drugs were polymerized as described below in the section on microtubule dynamics, but in the absence of rhodamine- or biotin-labeled tubulin. For protofilament number transition analysis, microtubules were polymerized from GMPCPP seeds with 1 mM GTP, 15 μM tubulin, 20 nM mCherry-EB3 in the presence of 100 nM Fchitax-3 at 37 °C for 10 min.

Sample preparation: 4 μl of each microtubule sample (unlabeled and labeled) was applied to holey carbon grids (C-flat 2/2, Protochips) glow-discharged in air, before blotting and plunge-freezing using a Vitrobot Mark IV (Thermo Fisher Scientific) at 22 °C and 100% humidity.

Cryo-EM data collection: For protofilament number distribution analysis, cryo-EM micrographs of Taxol and GMPCPP microtubules were collected on a Tecnai T12 transmission electron microscope (Thermo Fisher Scientific) with a 4x4K CCD camera (Gatan), operating at 120 kV, image pixel size of 2.09 Å and defocus of around -5 μm. Cryo-EM micrographs of Fchitax-3 microtubules were collected on a G2 Polara transmission electron microscope (Thermo Fisher Scientific) with a K2 Summit detector, operating in counting mode and with a GIF Quantum LS Imaging Filter (Gatan) at 300kV, image pixel size of 1.39 Å and defocus range from -1.5 to -4 μm. 40 frames were motion corrected using MotionCor2 (5). For protofilament number transition analysis, cryo-EM images of microtubules were collected on a Tecnai T12 (Thermo Fisher Scientific) as above.

Cryo-EM data analysis: For protofilament number distribution analysis, protofilament number was determined by Moiré pattern visualization. 50-187 microtubules were selected for each dataset. For each microtubule population, the percentage of microtubules with a particular protofilament number was calculated.

For protofilament number transition analysis, microtubule diameters on each side of sheet-like lattice defects in Fchitax-3-microtubules were measured manually in Fiji. Diameters of equivalently separated sides of intact microtubules were measured as controls. To aid diameter measurement, Fourier transforms of microtubule regions were computed and filtered to enhance microtubule Moiré patterns. The filtered Fourier transforms were then inverse transformed to give filtered microtubule images.

In vitro assay for microtubule dynamics

In vitro assay for microtubule growth dynamics was performed as described previously (6, 7). Briefly, as described earlier (3), microtubule seeds stabilized in the presence of GMPCPP (a slowly hydrolyzable GTP analog) were prepared by two rounds of polymerization and depolymerization in the presence of GMPCPP. A solution of 20 µM porcine brain tubulin mix containing 12% rhodamine-labeled tubulin/HiLyte 488^{TM} labeled tubulin and 18% biotin-labeled tubulin was polymerized in MRB80 buffer (80 mM K-PIPES, pH 6.8, 1 mM EGTA, 4 mM $MgCl_2$) in the presence of GMPCPP (1 mM) at 37 °C for 30 min. After polymerization, the mixture was pelleted by centrifugation at 119,000 × g for 5 min in an Airfuge centrifuge. Obtained pellet was resuspended in MRB80 buffer and microtubules were depolymerized on ice for 20 min. The resuspended mixture was further polymerized in the presence of GMPCPP. After the second round of polymerization and pelleting, GMPCPP-stabilized microtubule seeds were stored in MRB80 containing 10% glycerol. For preparing microtubule seeds in the presence of microtubule stabilizing agents (MSAs), a solution of porcine brain tubulin (40 µM) mix containing biotin-labeled tubulin (18%) and rhodamine-labeled tubulin (12%) was polymerized in MRB80 buffer (80 mM K-PIPES, pH 6.8, 4 mM $MgCl_2$, 1 mM EGTA) in the presence of GTP (1 mM) and indicated MSAs (20 µM) at 37 °C for 20 min. 20 µM MSAs (Taxol, Docetaxel, Alexa488-Epothilone B, Discodermolide and Fchitax-3) diluted in MRB80 were prewarmed to 37°C. Polymerizing tubulin solution was diluted 5 times with prewarmed 20 µM MSA solution and incubated further for 5 mins. The solution was centrifuged at 13200 × g for 15 min at 30 ^{0} C. Obtained pellet was resuspended in 20 µM MSA solution (diluted in MRB80) and stored at room temperature with protection from light.

In vitro flow chambers were assembled on microscopic slides with plasma-cleaned glass coverslips using two strips of double-sided tape. Flow chambers were sequentially incubated with PLL-PEG-biotin (0.2 mg/ml) and NeutrAvidin (1 mg/ml) in MRB80 buffer. The chamber was further incubated with GMPCPP stabilized microtubule seeds followed by treatment with 1 mg/ml κ-casein in MRB80 buffer. The reaction mixtures containing 15 µM porcine brain tubulin

supplemented with 3% rhodamine-tubulin when indicated, 20 nM mCherry-EB3 when indicated, 50 mM KCl, 0.1% methylcellulose, 0.2 mg/ml κ-casein, 1 mM guanosine triphosphate and oxygen scavenger mixture (50 mM glucose, 400 μg/ ml glucose oxidase, 200 μg/ml catalase, and 4 mM DTT in MRB80 buffer) with or without MSAs were added to the flow chambers after centrifugation in an Airfuge for 5 minutes at 119,000 × g. The chambers were sealed with vacuum grease, and microtubule dynamics was recorded using TIRF microscopy. All samples were imaged at 30 °C.

Image acquisition by TIRF microscopy

Imaging was performed on a TIRF microscope setup (inverted research microscope Nikon Eclipse Ti-E) which was equipped with the perfect focus system (PFS) (Nikon) and Nikon CFI Apo TIRF 100x 1.49 N.A. oil objective (Nikon, Tokyo, Japan). The microscope was supplemented with TIRF-E motorized TIRF illuminator modified by Roper Scientific France/PICT-IBiSA Institut Curie, and a stage top incubator model INUBG2E-ZILCS (Tokai Hit) was used to regulate the temperature of the sample. Image acquisition was performed using either a Photometrics Evolve 512 EMCCD camera (Roper Scientific, final magnification 0.063 μm/pixel) or a Photometrics CoolSNAP HQ2 CCD camera (Roper Scientific, final magnification 0.063 μm/pixel) or a Prime BSI sCMOS camera (Teledyne Photometrics, final magnification 0.068 μm/pixel) and controlled with MetaMorph 7.7 software (Molecular Devices, CA). For simultaneous imaging of red and green fluorescence, we used a triple-band TIRF polychroic ZT405/488/561rpc (Chroma) and a triple-band laser emission filter ZET405/488/561m (Chroma), mounted in a metal cube (Chroma, 91032) together with an Optosplit III beamsplitter (Cairn Research Ltd, Faversham, UK) equipped with double emission filter cube configured with ET525/50m, ET630/75m and T585LPXR (Chroma). Images were captured with 5 frames/s in stream acquisition mode for Fig. S2B, 10 frames/s, Fig. 5A, B, Fig. S10A, B upper panel, Fig. S11, 1 frame/s in time lapse mode for Fig. 3F and 1 frame/2s in time lapse mode for rest of the data.

Analysis of microtubule dynamics in vitro

For Image analysis, ImageJ plugin KymoResliceWide v.0.4 (https://github.com/ekatrukha/KymoResliceWide (Katrukha, 2015)) was used for generating kymographs to represent the life history of microtubule dynamics. Microtubule dynamics parameters using kymographs were measured manually. The length and the duration of each growth event were measured as horizontal and vertical distances on the kymograph, respectively. Microtubule growth rate was determined as a ratio of these values. Catastrophe frequency was calculated as the inverse growth time. We note that for the microtubule dynamics data analyzed here, calculating catastrophe frequency by dividing the total number of catastrophes by the total time microtubule spent in growth produced numbers that were very similar. Depolymerization events (shrinkage of plus end/loss of EB3 intensity) with the length 0.2-0.5 μm and "catching up" events were considered as short growth perturbation events. Short growth perturbations including

“catching up” events were considered as catastrophes when measuring catastrophe frequency. Depolymerization events shorter than 0.2 μm were not included in the analysis. We define dynamics as a stable rescue site (SRS dynamics) when a microtubule regrows at least 3 times from the same site after undergoing catastrophe. A random rescue is a single rescue event after a depolymerization episode that is longer than 0.5 μm. Two or more independent in vitro assays were performed for each reported experiment.

The values of critical concentrations and association rate constants (Fig.1E, S1E) were determined from the linear fit to the microtubule growth rate vs tubulin concentration points according to (8). To provide association rate constant values in micromolar units, we took into account different average protofilament number per condition (Fig.1A) and used conversion coefficient of 13*1000 nm/8 nm = 1625 tubulin dimers per micron for Taxol (13pf) (where one dimer length is 8 nm), 1750 for GMPCPP (14pf) and 1913 for Fchitax-3 (15.3pf) seeds respectively.

For analysis of microtubule dynamics after laser ablation, using ImageJ software, maximum projections were drawn, which provide the life history of the ablated microtubule fragments. Kymographs were drawn using ImageJ plugin KymoResliceWide v.0.4.

Quantification of EB3 and Fchitax-3 intensity time traces

For the analysis, experiments were performed with 15 μM tubulin, 20 nM mCherry-EB3 and 100 nM Fchitax-3 in the presence of GMPCPP seeds (mismatch conditions) or Fchitax-3 seeds (matching conditions). This analysis was performed similarly to the procedure described in (3), apart from time trace alignment. Briefly, simultaneous two-color imaging of Fchitax-3/mCherry-EB3 was performed using an OptoSplit III beamsplitter (Cairn Research Ltd, UK) equipped with double emission filter cube projecting two channels on the camera chip simultaneously. Chromatic aberrations were corrected as described previously using calibration photomask (9). Registered videos were used to create kymographs by drawing segmented lines of 10-15 pixel width (0.65-1 μm) along episodes of drug accumulation on growing microtubules using KymoResliceWide plugin with maximum transverse intensity (http://fiji.sc/KymoResliceWide). On extracted kymographs, we outlined rectangular regions of interest (ROI) around observed accumulation event and exported both intensities to MATLAB. For each time point, we fitted mCherry-EB3 profile with sum of constant (lattice binding) and exponential decay functions (comet) convoluted with microscope’s point spread function (PSF):

$$I(x) = I_{\mathrm{BG}} + \frac{1}{2} I_{\mathrm{lattice}} \cdot \mathrm{erfc}\left(\frac{x - x_{\mathrm{c}}}{\sqrt{2}\sigma_{\mathrm{PSF}}}\right) +$$

$$+ \frac{1}{2} I_{\mathrm{EB}} \cdot \exp\left(\frac{\lambda}{2}\left({\sigma_{\mathrm{PSF}}}^2 \lambda + 2(x - x_{\mathrm{c}})\right)\right) \cdot \left(1 - \mathrm{erf}\left(\frac{{\sigma_{\mathrm{PSF}}}^2 \lambda + x - x_{\mathrm{c}}}{\sqrt{2}\sigma_{\mathrm{PSF}}}\right)\right)$$

where fitting parameter I_{BG} corresponds to the intensity of background, $I_{lattice}$ to the amplitude of the fluorescence intensity fraction associated with the lattice binding, I_{EB} to the amplitude of convolved exponential decay, x_c to the position of the maximum number of molecules in the molecules distribution (start of exponential decay position), σ_{PSF} to the standard deviation of microscope's PSF and λ to the exponential decay constant. From the fitted function at each time frame we obtained maximum fluorescent intensity $I_{EB\_MAX}(t)$.

Intensity of Fchitax-3 was fitted using Gaussian function of variable width with the addition of background. Total intensity was calculated as an integrated area under the fitted curve (without background intensity) and provided $I_{Fchitax3}(t)$. Both $I_{EB\_MAX}(t)$ and $I_{Fchitax3}(t)$ intensity traces were normalized by the average of trace values above 80% percentile.

As a reference alignment, we chose Fchitax-3 channel for the time trace averaging, due to its clearly defined shape, and the EB3 time trace for each kymograph was shifted accordingly. Fchitax-3 traces were aligned using normalized cross-correlation. This pairwise function reports similarity between two temporal profiles depending on the displacement of one relative to another. It provides values in the range between zero and one, where higher values correspond to higher similarity. The global optimal alignment can be defined as a maximum of sum of cross-correlations between all profile pairs considering all possible displacements combinations. The number of combinations grows exponentially with the number of time traces and we found that time needed to explore the whole parameter space was too long for our datasets. Therefore, we devised an alternative algorithm searching for a local optimal alignment. We chose one time trace as a reference and registered all other time profiles to it, ensuring maximum of cross-correlation in each case. We repeated this procedure for all other time traces serving as a reference, and at the end chose the alignment with the maximum sum of cross-correlation functions among them. We observed that independent of the chosen reference profile, the majority of alignments provided very similar results with comparable cross-correlation score. This can be attributed to a distinctive sigmoid shape of Fchitax-3 temporal accumulation profile, making cross-correlation alignment converge to the same set of displacements. The corresponding MATLAB code performing the alignment and averaging, together with kymographs and ROIs used for the described analysis are available online (https://doi.org/10.6084/m9.figshare.c.5287663).

Quantification of microtubule tip tapering and EB3 comet intensity

Experiments were performed with GMPCPP seeds in the presence of 15 μM tubulin supplemented with 6.7% HiLyte 488™ labeled tubulin and 20 nM mCherry-EB3 (control, microtubule outgrowth from seeds) or the same mixture supplemented with 100 nM Discodermolide (mismatch conditions, microtubule outgrowth from stable rescue sites). Two-color imaging of HiLyte 488™ labeled tubulin and mCherry-EB3, chromatic aberration correction of movies and kymographs generation were performed in the same way as described in the previous section. EB3 comet traces during episodes of growth ending with a catastrophe were

manually outlined with polyline ROI, serving as initial estimation of a position. For each time point, intensity of HiLyte 488^{TM} labeled tubulin channel was fitted with the error function and background value:

$$I(x) = I_{\mathrm{BG}} + \frac{1}{2} I_{\mathrm{tubulin}} \cdot \mathrm{erf}\left(\frac{x - x_{\mathrm{c}}}{\sqrt{2}\sigma_{\mathrm{T}}}\right)$$

where fitting parameter I_{BG} corresponds to the intensity of background, $I_{tubulin}$ to the amplitude of the fluorescent signal along microtubule, x_c to the position of the microtubule tip and σ_{T} to the degree of tip tapering convolved with microscope's PSF. From each fitting we converted 95% confidence interval ΔC for the degree of tip tapering σ_{T} to the standard error (uncertainity) using formula $\Delta C/2t(0.5 * (1 + 0.95), DF)$, where t is Student's inverse cumulative distribution function and *DF* is number of degrees of freedom for the current fit. For the EB3 channel, maximum intensity of fitted comet $I_{EB\_MAX}(t)$ was obtained as described in the previous paragraph. EB3 comet time trace was normalized by the average peak intensity over last 20 seconds before the catastrophe after smoothing it with 2.5s interval. The corresponding MATLAB code, kymographs and ROIs used for this analysis are available online (https://doi.org/10.6084/m9.figshare.c.5287663).

Single molecule intensity analysis

Fchitax-3 molecules spontaneously binding to the coverslip during the reaction were used to measure single-molecule intensity (23). Briefly, two parallel flow chambers were made on the same coverslip. The first chamber was incubated with 100 nM Fchitax-3 without the reaction mixture. In second chamber, microtubule dynamics assay in the presence of Fchitax-3 stabilized microtubule seeds with 15 μM tubulin, 20 nM mCherry-EB3 and 100 nM Fchitax-3 was performed. For both the chambers, first 10-20 images of unexposed coverslip areas were acquired with the 100 ms exposure time using low laser intensity. In addition, a video of 300 frames exposing the same area with continuous laser illumination was recorded to induce photobleaching of Fchitax-3 molecules. Fluorescence intensities of Fchitax-3 molecules binding to the coverslip during reaction and in the solution of Fchitax-3 without the reaction mixture were detected and measured using ImageJ plugin DoM_Utrecht v.0.9.1 (https://github.com/ekatrukha/DoM_Utrecht). The fitted peak intensity values were used to build fluorescence intensity histograms. The intensities of Fchitax-3 molecules in the solution of Fchitax-3 without the reaction mixture and Fchitax-3 molecules transiently immobilized on the same coverslip during the reaction had the same intensity and showed the same single-step photobleaching behavior indicating Fchitax-3 molecules transiently immobilized to the coverslip during the reaction are single molecules. Further, integrated fluorescence intensities of Fchitax-3 molecules at the sites of drug accumulation during catch-up events in matching conditions were measured using ImageJ and were compared to Fchitax-3 single molecule intensity. These measurements were used to calculate the number of Fchitax-3 molecules at the drug

accumulation site. The number of Fchitax-3 molecules per 8 nm of microtubule length was calculated considering Fchitax-3-microtubules as 15pf.

Statistical analysis

GraphPad Prism 7 was used to plot all the histograms and statistical analysis was done using non-parametric Mann-Whitney U-test. For Fig. 4B, two-sided Fisher’s exact test was performed.

Supplementary Figure 1

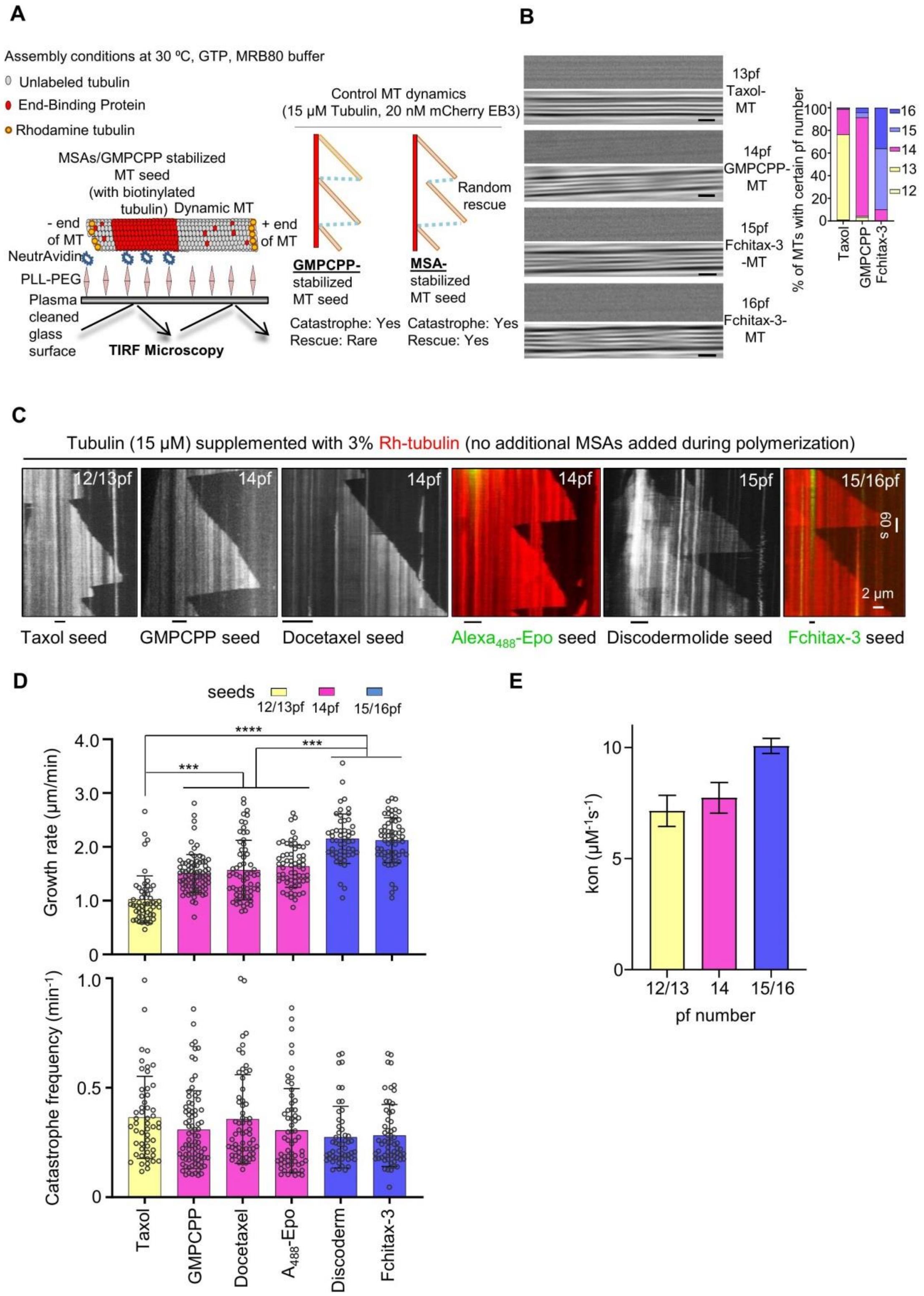

Fig. S1. Effects of microtubule protofilament number on microtubule growth rate.

A) Schematic representation of the in vitro microtubule dynamics assay and cartoons of kymographs illustrating microtubule growth events from a microtubule seed stabilized with GMPCPP or microtubule stabilizing agents (MSAs).

B) Representative raw cryo-EM images of labelled (rhodamine- and biotin-labeled tubulin) MTs (in the presence of Taxol, GMPCPP and Fchitax-3) and their filtered versions emphasizing Moiré patterns of certain types of microtubules. Scale bar, 25 nm. Bar graph shows microtubule protofilament number distribution for different conditions determined by Moiré pattern visualization for each microtubule. Microtubule population: n = 187 for Taxol; n = 95 for GMPCPP; n = 117 for Fchitax-3.

C) Representative kymographs showing microtubule dynamics in the presence of seeds (stabilized with GMPCPP or the indicated drugs) with different protofilament numbers in the presence of tubulin (15 μM supplemented with 3% of rhodamine tubulin) in the absence of EB3 and without additional MSAs added during the reaction.

D) Quantification of growth rates (D, upper panel) and catastrophe frequencies (D, lower panel) in the presence of seeds with different protofilament numbers as presented in panel C. From left to right, n = 52, 82, 62, 62, 51, 60 growth events. N = 3 independent experiments for Taxol, GMPCPP and Fchitax-3- microtubule seeds, N = 2 independent experiments for Docetaxel and Alexa488-Epothilone B- microtubule seeds, error bars represent SD; ***, p <0.001, ****, p <0.0001, Mann-Whitney U test.

E) Tubulin association rate constants determined from the slope of the plots of the growth rate vs tubulin concentration (Fig. 1E) for the microtubules grown from seeds with different protofilament number.

Supplementary Figure 2

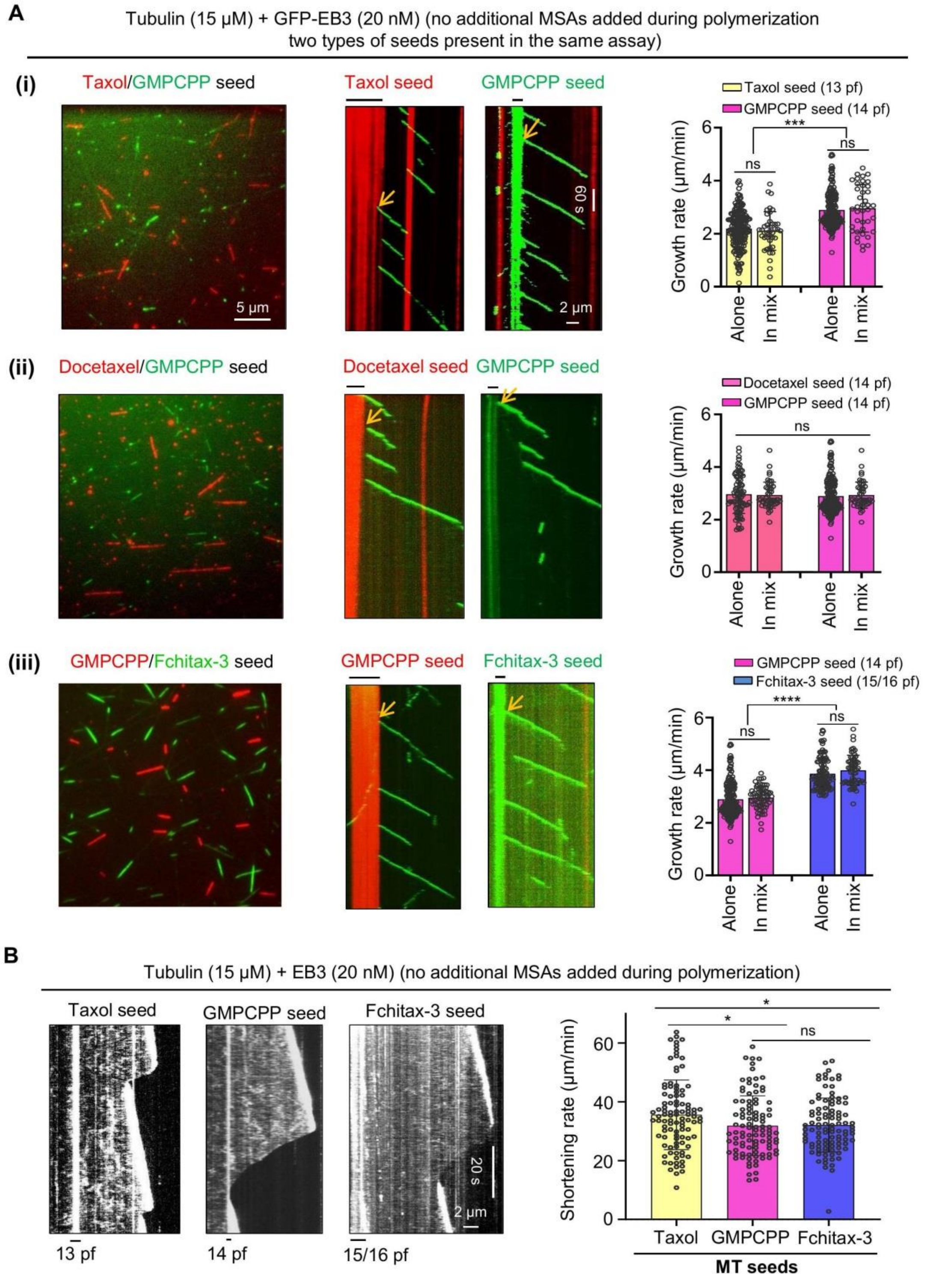

Fig. S2. Effects of microtubule protofilament number on microtubule growth and depolymerization rates.

A) Still images and representative kymographs showing the presence of two different kinds of seeds and microtubules growing from these seeds in the same assay. In the upper panel, Taxol (12/13pf) and GMPCPP (14pf) seeds were mixed (i). In the middle panel, Docetaxel seeds (14pf) were mixed with GMPCPP seeds (14pf) (ii). In the bottom panel GMPCPP seeds (14pf) were mixed with Fchitax-3 seeds (15/16pf) (iii). Respective bar graphs show the quantification of microtubule growth rate from different seeds during seed mixing conditions. Only growth events originating from seeds (highlighted by yellow arrows) and not from rescue sites were included in the quantification. For better comparison, data from Figure 1D ("alone") are included in the present panels. From left to right- n = 193, 40, 196, 40 (panel i), n = 82, 47, 196, 47 (panel ii), n = 196, 66, 104, 60 (panel iii).

B) Representative kymographs showing microtubule depolymerization events as indicated. Bar graph shows the quantification of shortening rates. N = 3 independent experiments, n = 100 for all the conditions, *, $p < 0.01$, Mann-Whitney U test.

Supplementary Figure 3

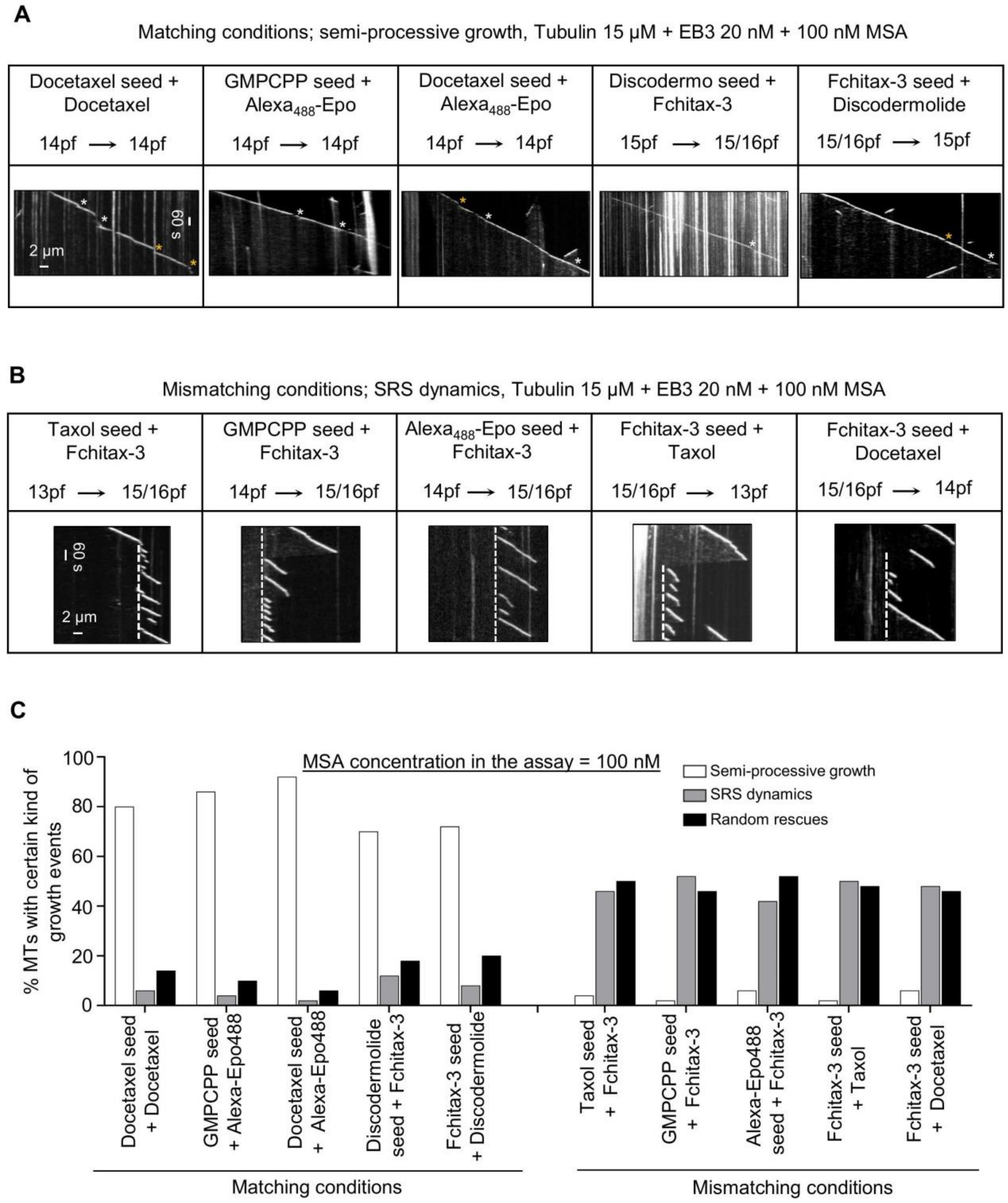

Fig. S3. Effects of protofilament number mismatch between the seed and growth conditions on microtubule dynamics.

A, B) Representative kymographs showing microtubule dynamics in the indicated conditions. N = 3 independent experiments. In matching conditions, short growth perturbation events followed by rapid rescues are highlighted (white asterisks highlight split comets and yellow asterisks highlight

depolymerization events with the length 0.2-0.5 μm). In SRS dynamics, stable rescue sites in mismatching conditions are highlighted by white stippled lines.

C) Quantification of microtubule growth patterns for conditions shown in panel A and B. n = 50 microtubule seeds in all conditions.

Supplementary Figure 4

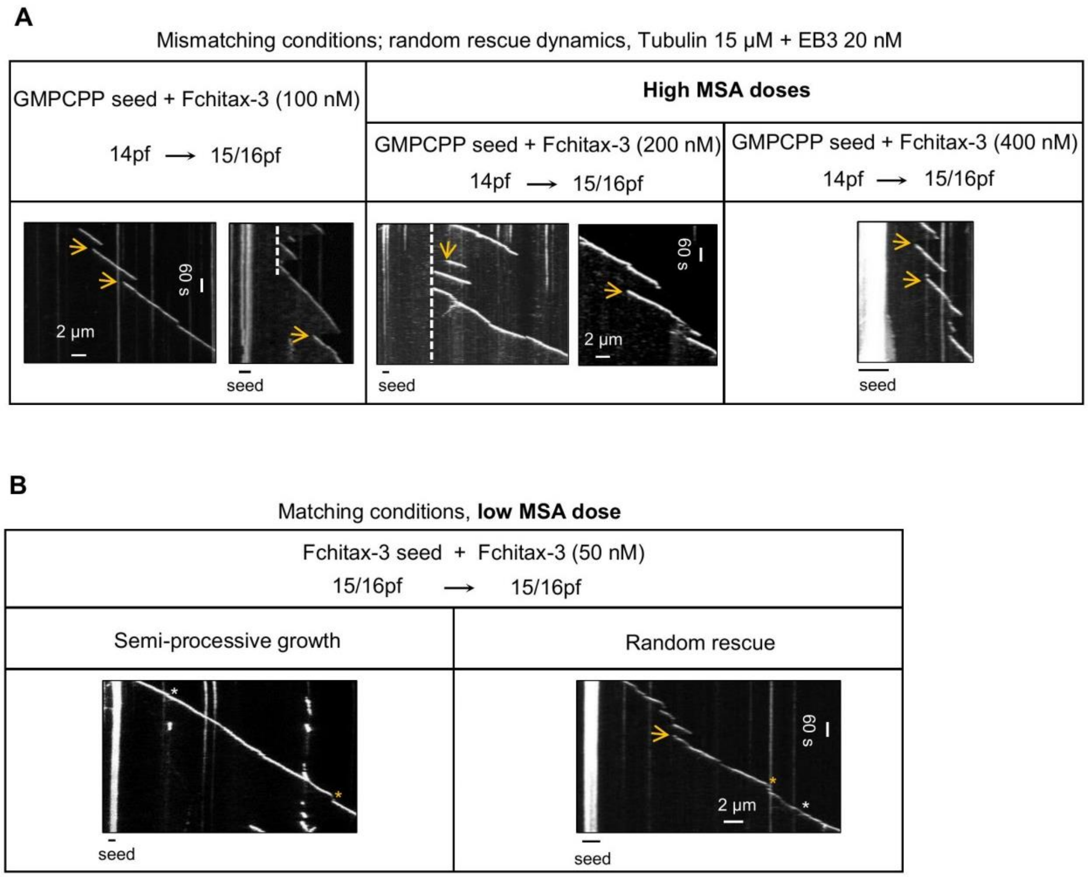

Fig. S4. Effects of protofilament number mismatch between the seed and growth conditions on microtubule dynamics at different MSA concentrations.

A, B) Representative kymographs illustrating microtubule dynamics in different conditions, as indicated. Random rescues are indicated with yellow arrows, white stripped lines highlight SRS, white asterisks highlight split comets and yellow asterisks highlight depolymerization events with the length 0.2-0.5 μm.

Supplementary Figure 5

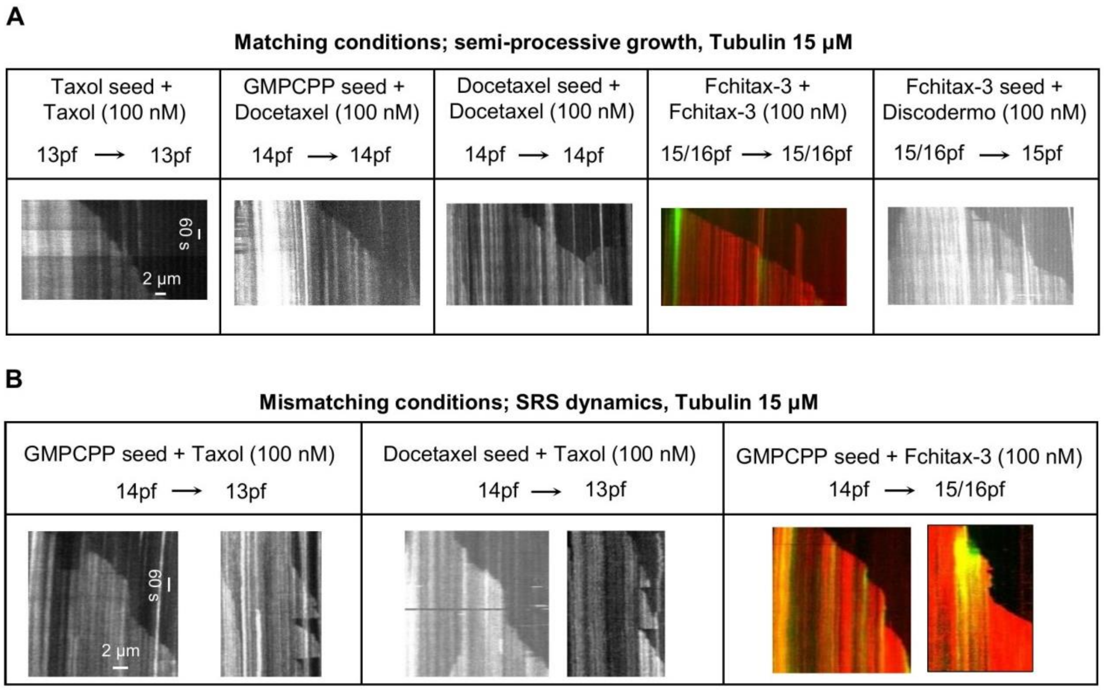

Fig. S5. Effects of protofilament number mismatch between the seed and growth conditions on microtubule dynamics in the absence of EB3.

A, B) Kymographs showing microtubule dynamics in the absence of EB3 for microtubules polymerized in the indicated conditions.

Supplementary Figure 6

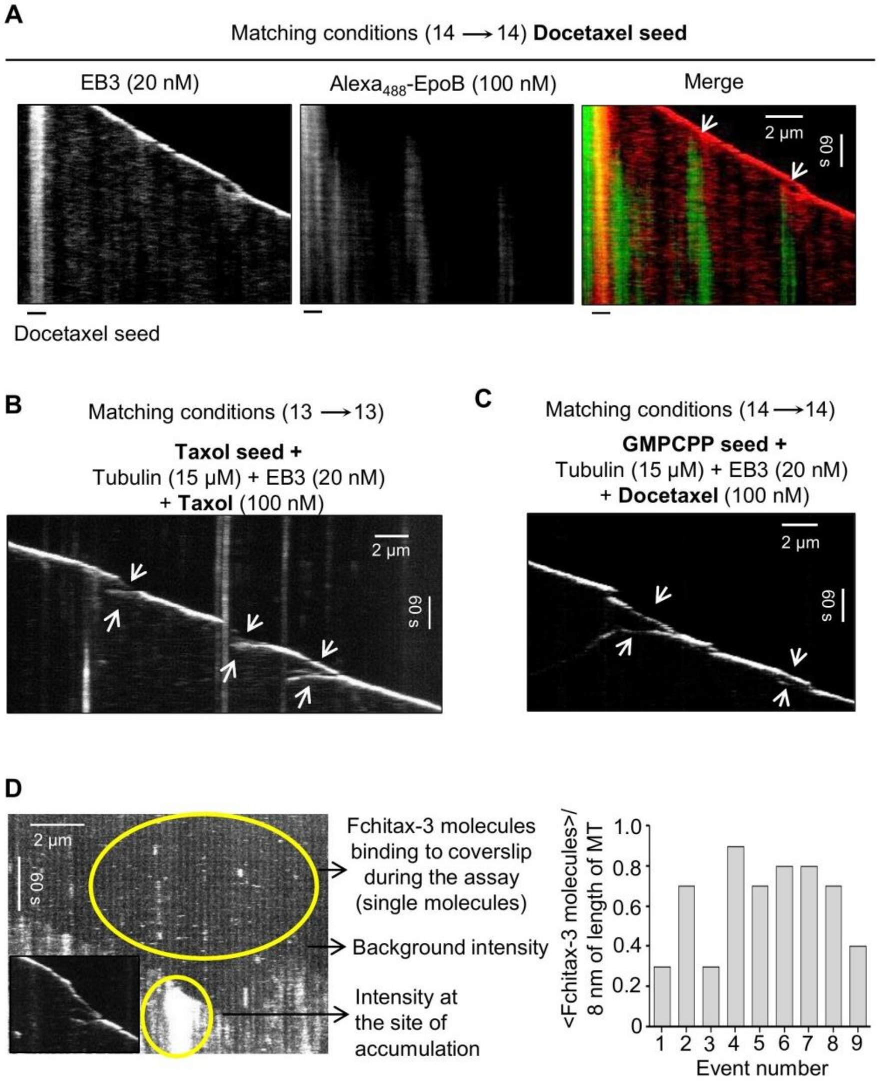

Fig. S6. Characterization of microtubule growth perturbations in the presence of MSAs in matching conditions.

A) Representative kymographs illustrating the dynamics of microtubules grown in matching conditions, from Docetaxel seeds in the presence of 15 µM tubulin and 20 nM mCherry-EB3 with Alexa$_{488}$-Epothilone B (100 nM). White arrows indicate split comets.

B, C) Representative kymographs showing split comets (“catching up” events, white arrows) in matching conditions as indicated.

D) Kymograph illustrating the accumulation of Fchitax-3 molecules at the site of a split comet (inset) and free Fchitax-3 molecules binding to coverslip during the assay. Quantification of the number of Fchitax-3 molecules at the accumulation site is shown on the right.

Supplementary Figure 7

A

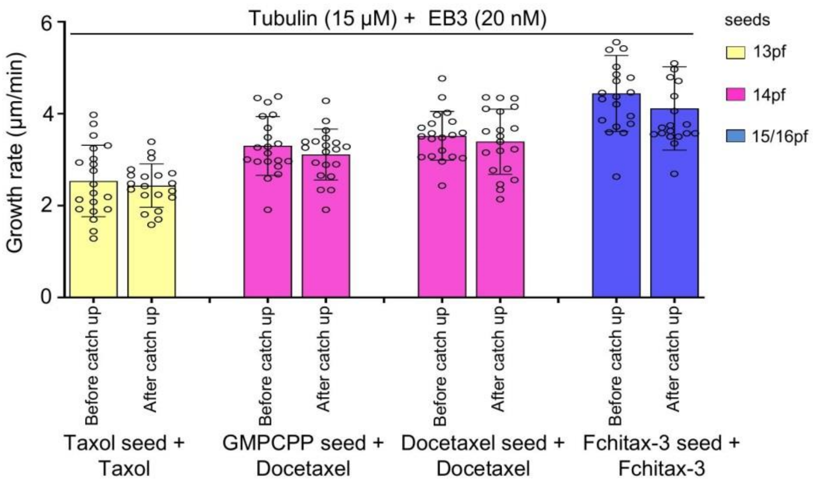

B

Intact MT				MT with a defect			
Thinner side diameter (Mean ± sd, nm)	Thicker side diameter (Mean ± sd, nm)	Mean diameter difference (nm)	N:diff < 2 nm Y:diff > 2 nm	Thinner side diameter (mean ± sd, nm)	Thicker side diameter (Mean ± sd, nm)	Mean diameter difference (nm)	N:diff < 2 nm Y:diff > 2 nm
27.0 ± 0.3	27.5 ± 0.2	0.5	N	25.6 ± 0.5	26.6 ± 0.3	1.0	N
26.3 ± 0.6	26.9 ± 0.4	0.6	N	27.5 ± 0.2	28.1 ± 0.2	0.6	N
26.7 ± 1.1	27.3 ± 0.3	0.6	N	27.9 ± 0.3	30.4 ± 1.3	2.5	Y
25.5 ± 0.8	26.4 ± 0.7	0.9	N	26.8 ± 0.4	29.9 ± 0.3	3.1	Y
26.3 ± 0.7	26.6 ± 0.8	0.3	N	20.9 ± 0.4	26.7 ± 0.1	5.8	Y
26.9 ± 0.5	27.3 ± 0.2	0.4	N	27.8 ± 0.5	28.0 ± 0.2	0.2	N
26.9 ± 0.6	27.0 ± 0.7	0.1	N	26.4 ± 0.3	27.9 ± 0.3	1.5	N
25.9 ± 1.0	26.0 ± 0.8	0.1	N	26.1 ± 0.9	29.5 ± 0.7	3.4	Y
26.6 ± 0.7	26.9 ± 0.6	0.3	N	25.3 ± 0.7	26.4 ± 0.8	1.1	N
27.1 ± 0.5	27.1 ± 0.7	0.0	N	27.2 ± 0.2	28.7 ± 0.4	1.5	N
27.1 ± 0.5	27.1 ± 1.2	0.0	N	24.3 ± 0.4	25.8 ± 0.9	1.5	N
26.6 ± 0.3	27.3 ± 0.8	0.7	N	24.2 ± 0.1	26.2 ± 0.5	2.0	Y
27.2 ± 0.4	27.3 ± 0.2	0.1	N	22.8 ± 0.3	26.3 ± 1.0	3.5	Y
27.6 ± 0.3	27.6 ± 0.8	0.0	N	25.0 ± 0.3	28.6 ± 0.6	3.6	Y
26.9 ± 0.3	26.9 ± 0.5	0.0	N	26.1 ± 0.4	32.5 ± 0.6	6.4	Y
27.7 ± 1.0	27.8 ± 0.2	0.1	N	26.5 ± 0.2	27.0 ± 0.4	0.5	N
28.1 ± 0.2	28.8 ± 0.3	0.7	N	27.1 ± 0.5	30.1 ± 0.8	3.0	Y
26.3 ± 0.4	27.3 ± 0.2	1.0	N				
27.0 ± 0.7	29.5 ± 0.7	2.5	Y				

Fig. S7. Analysis of changes in protofilament number associated with split comets and lattice defects.

A) Quantification of growth rates before and after a “catching up” event in matching conditions. 20 events were analyzed per condition.

B) A table showing the comparison of microtubule diameter on either side of a defect in microtubules grown from GMPCPP seeds in the presence of Fchitax-3. Microtubules with diameter difference of ≥ 2 nm were identified as microtubules with protofilament number transition. All possible microtubules with defects were picked from the dataset, excluding those with ice contamination and in crowded areas of the micrographs; as a control, an equivalent number of microtubules without defects were picked at random, also excluding those with ice

contamination and in crowded areas of the micrographs. We used Fisher's exact test, which is appropriate for the N of our data, to assess the difference in protofilament transition frequency in these microtubules.

Supplementary Figure 8

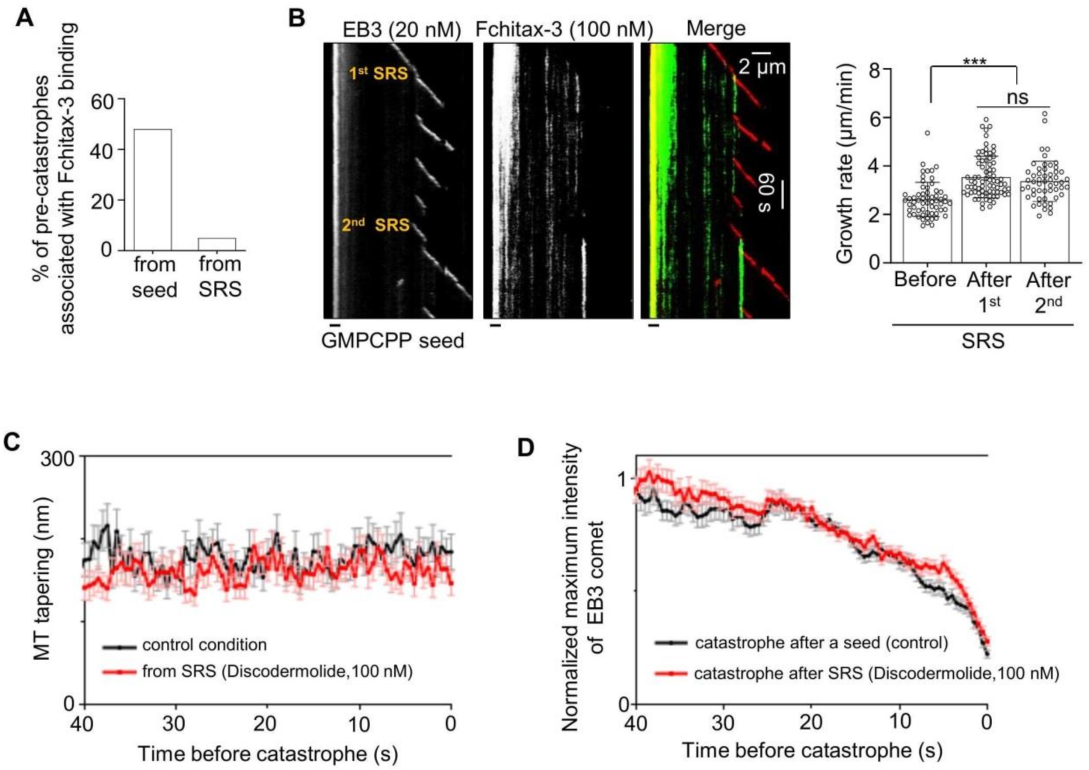

Fig. S8. Analysis of drug accumulations at pre-catastrophe microtubule ends and microtubule tip tapering.

A) Quantification of frequency of drug accumulations at pre-catastrophe microtubule ends (identified by the strong reduction or complete loss of EB3 intensity, as shown in Figure 3F) growing either from a seed or after a stable rescue site (SRS).

B) Representative kymographs showing occurrence of a second stable rescue site (SRS) after the formation of the first SRS within the same microtubule. The experiment was performed with GMPCPP seeds in the presence of 15 μM tubulin, 20 nM EB3 and 100 nM Fchitax-3. Bar graph shows quantification of microtubule growth rates before (n = 60) and after the first (n = 75) and the second (n = 53) stable rescue site. N = 3 independent experiments, ns = not significant, ***, p <0.001, Mann-Whitney U test.

C, D) The degree of microtubule tip tapering (C) (derived from the fit to error function, with higher values corresponding to higher tapering) and averaged normalized maximum intensity of fitted EB3 comet (D) aligned by the moment of catastrophe. Black curves correspond to microtubules growing from seeds in control conditions (15 μM tubulin supplemented with 6.7% green tubulin, 20 nM mCherry-EB3 in the presence of GMPCPP seeds, 73 kymographs from 5 experiments). Red curves correspond to growth events from stable rescue sites (15 μM tubulin supplemented

with 6.7% green tubulin, 20 nM mCherry-EB3 and 100 nM Discodermolide in the presence of GMPCPP seed, 64 kymographs from 5 experiments). Error bars represent SEM. The average uncertainty of fitting for the degree of tapering parameter was equal to 56.3±18.9 nm (±SD).

Supplementary Figure 9

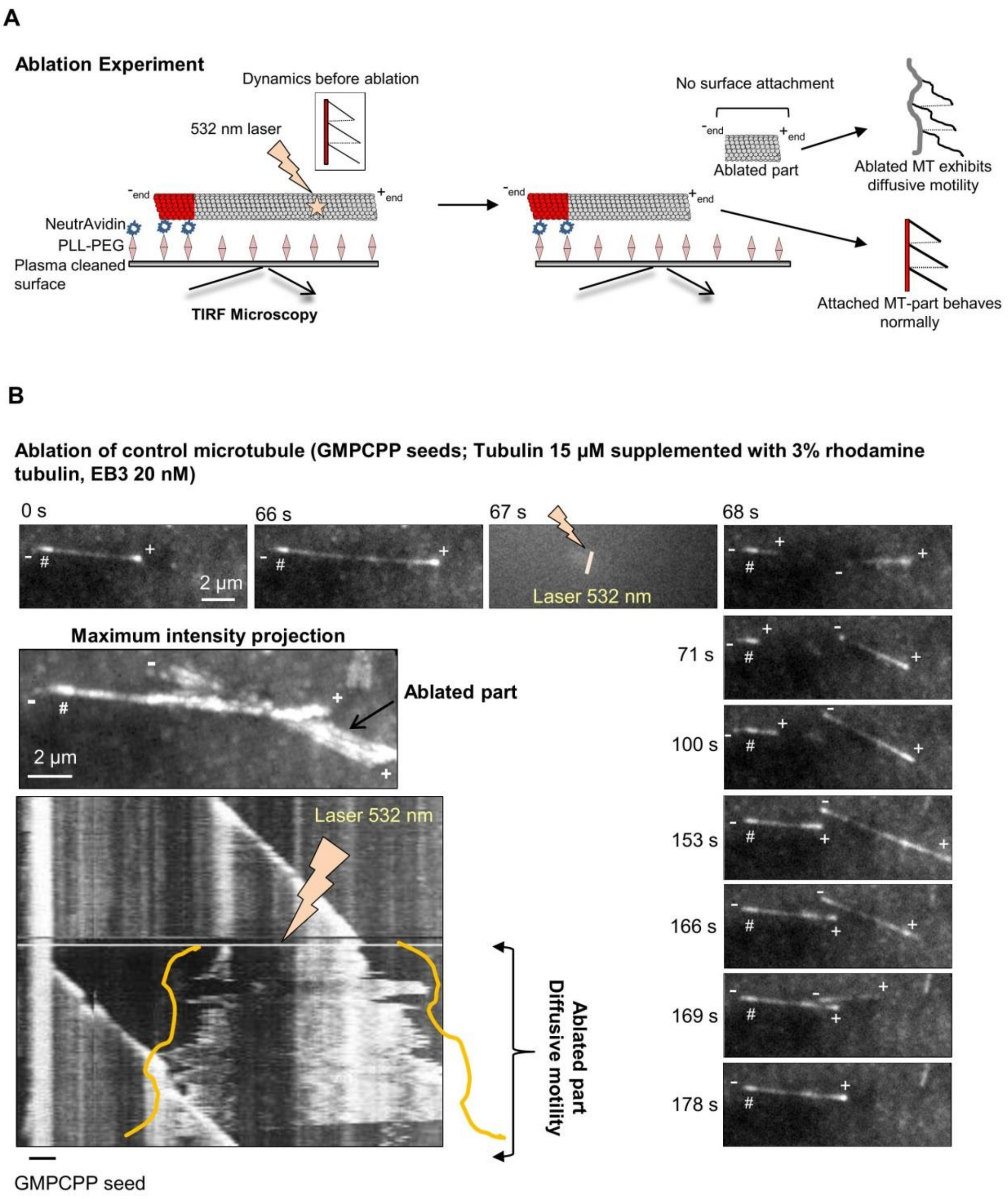

Fig. S9. Laser mediated microtubule severing.

A) A cartoon depicting laser (532 nm) ablation experiments with a TIRF-microscopy setup. The severed microtubule fragment is not attached to the coverslip surface and shows some diffusive motility.

B) Still images showing photoablation of a control microtubule. The assay was performed in the presence of GMPCPP seeds with 15 µM tubulin, supplemented with 3% rhodamine-tubulin and 20 nM mCherry-EB3. # shows the position of the GMPCPP seed; the site of laser ablation is indicated by a lightning bolt. The plus and minus ends of the microtubule growing from the

GMPCPP seed and the newly generated fragment are highlighted. The severed microtubule fragment is not attached to the coverslip surface and shows some diffusive motility, but stays in the same focal plane, as illustrated by a maximum intensity projection generated using ImageJ software. Kymographs were drawn using ImageJ plugin KymoResliceWide v.0.4.

Supplementary Figure 10

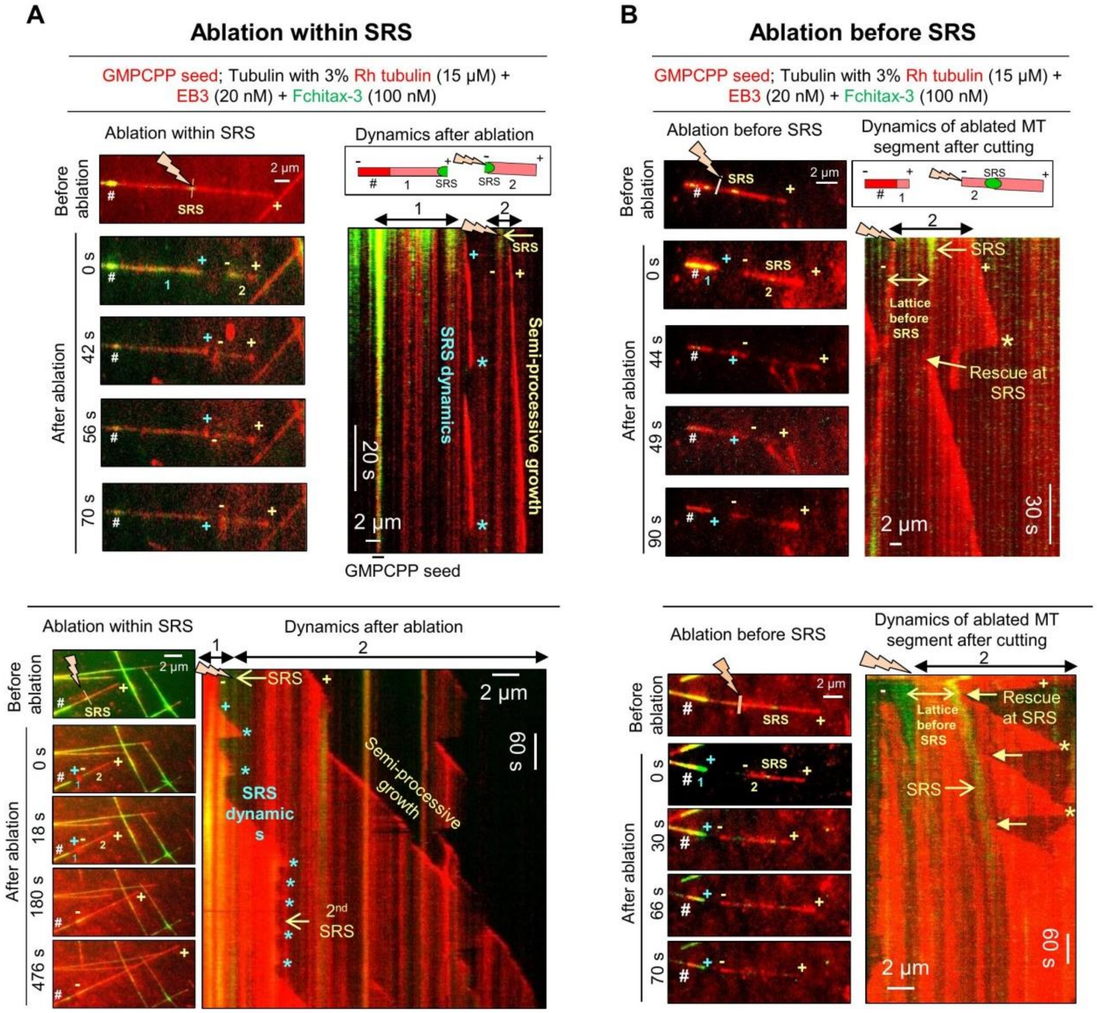

Fig. S10. Microtubule severing within or before stable rescue site.

A, B) Still images showing microtubule photoablation within (A) or before (B) a drug accumulation area (SRS) and kymographs illustrating microtubule dynamics of the severed microtubule segment after cutting. Upper panels, examples with faster acquisition (10 frames/s, stream acquisition). Lower panels, examples with slow acquisition (time-lapse acquisition with 1 frame/2s). The frame before ablation highlights the position of the GMPCPP seed (#), the plus (+) end of the microtubule, the position of Fchitax-3 accumulation within microtubule (SRS) and the site of laser ablation within or before SRS. After ablation, the severed microtubule parts (highlighted by 1 and 2), microtubule plus ends and the new ends generated after ablation are indicated. In panel A, kymographs illustrate the dynamics of both fragments (1 and 2) generated after ablation (as shown in the scheme). In panel B, kymographs illustrate the dynamics of the severed microtubule fragments (2, as shown in the scheme). Asterisks indicate catastrophes; rescues at the stable rescue site within the severed part are indicated by arrows in panel B. The

labels are in blue for fragment 1 (seed-attached microtubule part) and yellow for fragment 2 (the part detached after ablation).

Supplementary Figure 11

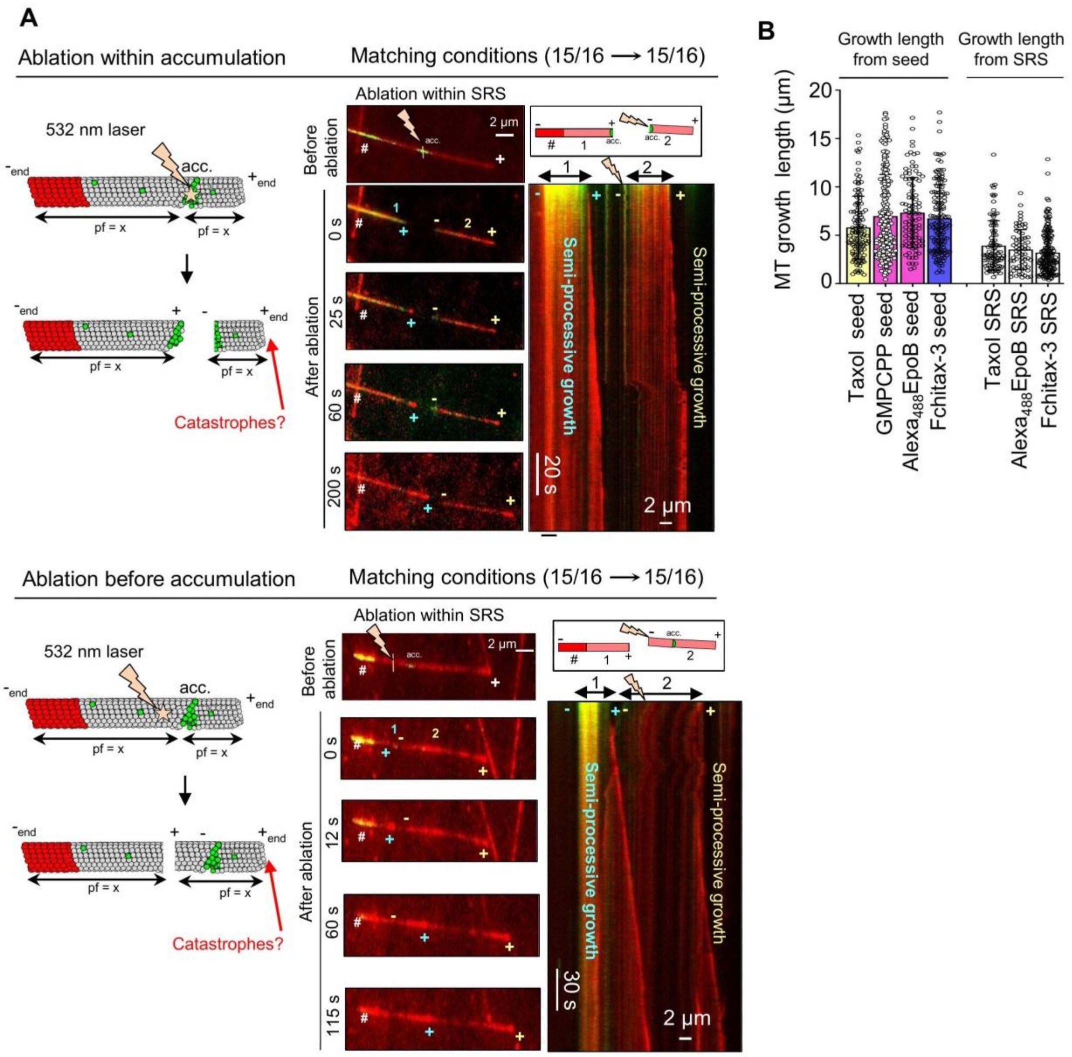

Fig. S11. Microtubule severing in matching conditions and analysis of microtubule growth length.

A) A cartoon and still images showing microtubule photoablation within (upper panel) or before (lower panel) a drug accumulation area in matching conditions (Fchitax-3 seeds in the presence of 15 µM tubulin supplemented with 3% rhodamine tubulin, 20 nM EB3 and 100 nM Fchitax-3). The frame before ablation highlights the position of the GMPCPP seed (#), the plus (+) end of the microtubule, the position of Fchitax-3 accumulation within microtubule and the site of laser ablation within or before accumulations. After ablation, the severed microtubule parts (indicated by 1 and 2), microtubule plus ends and the new ends generated after ablation are indicated. Kymographs illustrate microtubule dynamics of both the newly generated plus end (seed-attached microtubule part; fragment 1 and the part detached after ablation; fragment 2). Images were

acquired at 10 frames/s, with stream acquisition. The labels are in blue for fragment 1 (seed-attached microtubule part) and yellow for fragment 2 (the part detached after ablation).

B) Analysis of microtubule growth length for different conditions as indicated. From left to right: n = 118, 201, 96, 140, 92, 65 and 170.

Legends to Supplementary Videos

Video S1. Laser severing of control microtubules.

The video illustrates photoablation of a control microtubule as depicted in Figure S8B. The experiment was performed in the presence of GMPCPP seeds with 15 µM tubulin supplemented with 3% rhodamine-tubulin and 20 nM mCherry-EB3. Laser ablation was performed using 532 nm laser on a TIRF setup. The video was acquired with a 1 s interval between frames and an exposure time of 100 ms. At the start, the positions of GMPCPP seed (#), plus (+) and minus (-) ends of the microtubule are shown. The moment and the site of ablation (!) are also marked at 67 s. The newly generated ends of the ablated part and the pre-existing ends are highlighted at 69 s. Scale bar, 2 µm. The video is representative of 5 independent experiments.

Video S2. Laser severing of microtubule lattice within the Fchitax-3 accumulation zone (stable rescue site).

The video illustrates photoablation of a microtubule within the Fchitax-3 accumulation zone (SRS), as depicted in Figure 5A. The experiment was performed in the presence of GMPCPP seeds with 15 µM tubulin supplemented with 3% rhodamine-tubulin, 20 nM mCherry-EB3 and 100 nM Fchitax-3. The video first shows a frame before photoablation, which highlights the position of the GMPCPP seed (#), the plus (+) end of the microtubule, the site of Fchitax-3 accumulation (SRS) and the site of laser ablation (!) within SRS. After photoablation within the SRS region, the video was recorded in a stream acquisition mode with an exposure time of 100 ms. After photoablation, only the red channel is presented. At the start of the image sequence after photoablation (0 s), the positions of the GMPCPP seed (#) and the microtubule ends are indicated. At 97.2 s, the growing ends of both parts are indicated. At 103.8 s, a catastrophe (*) of the seed-attached microtubule lattice is indicated. Scale bar, 2 µm. The video is representative of 5 independent experiments.

Video S3. Laser severing of microtubule lattice before Fchitax-3 accumulation zone (stable rescue site).

The video illustrates photoablation of a microtubule before the Fchitax-3 accumulation zone (SRS) as depicted in Figure 5B. The experiment was performed in the presence of GMPCPP seeds with 15 µM tubulin supplemented with 3% rhodamine-tubulin, 20 nM mCherry-EB3 and 100 nM Fchitax-3. The video first shows a frame before photoablation, which highlights the position of the GMPCPP seed (#), plus (+) end of the microtubule, the plus (+) end of the microtubule, the site of Fchitax-3 accumulation (SRS) and the site of laser ablation (!) before SRS. After photoablation, the video was recorded in a stream acquisition mode with an exposure time of 100 ms. After photoablation, only tubulin channel is presented. At the start of the image sequence after photoablation (0 s), the positions of the GMPCPP seed (#), and the microtubule ends are highlighted. After photoablation, both microtubule parts start growing (highlighted at 8.4 s and

66.4s). A catastrophe (*) of the ablated part is highlighted at 67.2 s. Scale bar, 2 µm. The video is representative of 5 independent experiments.

SI References

1. Diaz JF & Andreu JM (1993) Assembly of purified GDP-tubulin into microtubules induced by taxol and taxotere: reversibility, ligand stoichiometry, and competition. *Biochemistry* 32(11):2747-2755.
2. Li X, Barasoain I, Matesanz R, Diaz JF, & Fang WS (2009) Synthesis and biological activities of high affinity taxane-based fluorescent probes. *Bioorg Med Chem Lett* 19(3):751-754.
3. Rai A*, et al.* (2020) Taxanes convert regions of perturbed microtubule growth into rescue sites. *Nat Mater* 19(3):355-365.
4. Paterson I, Florence GJ, Gerlach K, Scott JP, & Sereinig N (2001) A practical synthesis of (+)-discodermolide and analogues: fragment union by complex aldol reactions. *J Am Chem Soc* 123(39):9535-9544.
5. Zheng SQ*, et al.* (2017) MotionCor2: anisotropic correction of beam-induced motion for improved cryo-electron microscopy. *Nat Methods* 14(4):331-332.
6. Bieling P*, et al.* (2007) Reconstitution of a microtubule plus-end tracking system in vitro. *Nature* 450(7172):1100-1105.
7. Mohan R*, et al.* (2013) End-binding proteins sensitize microtubules to the action of microtubule-targeting agents. *Proc Natl Acad Sci U S A* 110(22):8900-8905.
8. Walker RA*, et al.* (1988) Dynamic instability of individual microtubules analyzed by video light microscopy: rate constants and transition frequencies. *J Cell Biol* 107(4):1437-1448.
9. Aher A*, et al.* (2018) CLASP Suppresses Microtubule Catastrophes through a Single TOG Domain. *Dev Cell* 46(1):40-58.
